# Dynamics of Metabolic Pathways and Stress Response Patterns during Human Neural Stem Cell Proliferation and Differentiation

**DOI:** 10.3390/cells11091388

**Published:** 2022-04-20

**Authors:** Vesselina Semkova, Simone Haupt, Michaela Segschneider, Catherine Bell, Magnus Ingelman-Sundberg, Mohamad Hajo, Beatrice Weykopf, Pathma Muthukottiappan, Andreas Till, Oliver Brüstle

**Affiliations:** 1 Institute of Reconstructive Neurobiology, Medical Faculty & University Hospital Bonn, University of Bonn, 53127 Bonn, Germany; 2 LIFE & BRAIN GmbH, Cellomics Unit, 53127 Bonn, Germany; 3 Karolinska Institute, Department of Physiology and Pharmacology, 171 77 Stockholm, Sweden

**Keywords:** neurodevelopment, neuronal differentiation, NRF2, autophagy, ROS

## Abstract

Understanding early nervous system stress response mechanisms is crucial for studying developmental neurotoxicity and devising neuroprotective treatments. We used hiPSC-derived long-term self-renewing neuroepithelial stem (lt-NES) cells differentiated for up to 12 weeks as an in vitro model of human neural development. Following a transcriptome analysis to identify pathway alterations, we induced acute oxidative stress (OS) using tert-butyl hydroperoxide (TBHP) and assessed cell viability at different stages of neural differentiation. We studied NRF2 activation, autophagy, and proteasomal function to explore the contribution and interplay of these pathways in the acute stress response. With increasing differentiation, lt-NES cells showed changes in the expression of metabolic pathway-associated genes with engagement of the pentose phosphate pathway after 6 weeks, this was accompanied by a decreased susceptibility to TBHP-induced stress. Microarray analysis revealed upregulation of target genes of the antioxidant response KEAP1–NRF2–ARE pathway after 6 weeks of differentiation. Pharmacological inhibition of NRF2 confirmed its vital role in the increased resistance to stress. While autophagy was upregulated alongside differentiation, it was not further increased upon oxidative stress and had no effect on stress-induced cell loss and the activation of NRF2 downstream genes. In contrast, proteasome inhibition led to the aggravation of the stress response resulting in decreased cell viability, derangement of NRF2 and KEAP1 protein levels, and lacking NRF2-pathway activation. Our data provide detailed insight into the dynamic regulation and interaction of pathways involved in modulating stress responses across defined time points of neural differentiation.

## 1. Introduction

There is accumulating evidence that the exposure of embryonic brain cells to environmental toxins is associated with deleterious effects on embryonic health [1,2]. A comprehensive analysis of biomonitoring data in 2011 revealed at least 43 different environmental chemicals to be present in the body of every pregnant woman in the US [3]. Many of these pollutants, including polychlorinated biphenyls, methylmercury, and different pesticides, are known inducers of oxidative stress [4,5,6,7], and epidemiological studies have linked exposure to these agents with impaired neurodevelopment in children [8,9,10,11,12,13]. To unravel the molecular events underlying these observations, analyzing neurodevelopmental mechanisms of toxicity in vitro is necessary. Such analysis would facilitate the risk assessment process and identify possible environment–gene interactions that lead to neurodevelopmental disorders or could mediate their prevention [14]. Along this line, human pluripotent stem cell (hPSC) technology, in combination with targeted differentiation approaches, has the potential to meet the increasing need for alternative models to study and test for developmental neurotoxicity events. However, detailed characterization of the stress response mechanisms in these systems is still missing and represents an important stepping stone on the road to benefit from their versatile applications.

Cellular defense pathways such as the ubiquitous nuclear factor (erythroid-derived 2)-like-2 factor (NRF2) antioxidant pathway are frontline protection against oxidative stress caused by organic pollutants [15,16,17]. NRF2 is a basic leucine zipper transcription factor, which exerts its action by translocating to the nucleus and mediating the expression of a battery of cytoprotective genes. These genes carry a specific sequence called the antioxidant response element (ARE) in their promoter regions [18]. Under homeostatic conditions, NRF2 is anchored to its negative regulator kelch like ECH associated protein 1 (KEAP1), a cysteine-rich protein that sequesters NRF2 in the cytoplasm thus preventing its transcriptional activity. Under oxidative stress conditions, KEAP1 plays the role of a redox-sensitive switch and, after oxidation-dependent modification of critical amino acids, releases NRF2, thus enabling its nuclear translocation and activation of the downstream gene expression program [19,20]. NRF2 expression has been shown to be shut off during early in vitro neuronal development. This contributes to weakened antioxidant defense capacity during this critical phase allowing key developmental pathways which depend on reactive oxygen species (ROS) signaling to become activated [21].

The differentiation of a precursor cell into a neuron is a process encompassing major changes in cell morphology and remodeling of subcellular processes. A central hallmark of differentiation is a shift in energy metabolism from glycolysis to oxidative phosphorylation (OXPHOS) accompanied by mitochondrial maturation [22,23,24]. However, higher ATP energy yields come at a price, with mitochondrial metabolism resulting in a dramatic increase in the endogenous ROS levels as a by-product. Interestingly, oxidative stress is an integral part of normal neurogenesis and contributes to regulating redox-sensitive pathways such as PI3K/Akt, WNT, and JNK signaling [21,25,26]. Therefore, tight regulation of endogenous antioxidant pathways is necessary to keep the delicate balance between physiological and pathogenic stress conditions.

In addition to dynamic alterations in redox responses, neuronal differentiation is tightly coupled to remodeling proteostasis. Neurons are highly polarized cells, and during development they undergo extensive morphological changes, including axonal branching and synapse formation, both requiring a dynamic rearrangement in the cellular cytoskeletal system and reshaping of cellular compartments and trafficking events [27,28]. Regulated protein elimination via the two main eukaryotic proteolysis systems, autophagy [29,30,31] and the ubiquitin–proteasome system (UPS) [32,33,34], contributes to brain development by regulating different molecular mechanisms such as axon guidance, axon and dendritic branching, synaptogenesis and establishment of synaptic connections. Proteostasis also plays an important role under elevated ROS conditions by regulating the clearance of oxidized and damaged cellular components, and a functional interplay between NRF2 and both autophagy and proteasomal degradation has been identified [35,36,37,38,39,40,41].

In the present study, we used a stable population of long-term self-renewing rosette-type human iPSC-derived neural stem (lt-NES) cells as a reductionist model of human neural in vitro development. Following growth factor withdrawal, lt-NES cells become postmitotic and give rise to large numbers of neurons. With prolonged differentiation, glial cells emerge as well [42,43]. Differentiated lt-NES cells have been used extensively for modeling neuronal degeneration [44,45,46]. Here, we studied how neurodevelopmental progression from proliferating lt-NES cells to their differentiated neural progeny affects the regulation of antioxidant and proteostatic stress responses. We explored changes in gene expression during differentiation, which indicate alterations in cell metabolism and NRF2 pathway activity. These events were accompanied by increasing resistance to ectopic stress with culture maturation. The NRF2 pathway showed dynamic modulation from the proliferative throughout the differentiated states, and its activity contributed to the increasing resistance to stress. We further assessed components of the proteostatic machinery in proliferative and differentiated lt-NES cells. The autophagic pathway was upregulated with differentiation but appeared to play no frontline role in acute stress response. Although basal proteasomal activity was not altered with differentiation, the proteasome was indispensable in the acute response to stress by regulating the NRF2 pathway. These data further add to our current understanding of the dynamic changes in stress responses that occur during early human neurodevelopment.

## 2. Materials and Methods

### 2.1. Ethics Statement

The cell line used in this study was generated from donor sample-derived iPS cells, which were obtained after signing written informed consent. IPS cell generation was approved by the Ethics Committee of the Medical Faculty, University of Bonn (permit number: 275/08).

### 2.2. lt-NES Cell Culture Maintenance and Differentiation

lt-NES cells were generated as previously described [43], using the retrovirally transformed fibroblast-derived iPS cell line 31fr1. The cells were maintained on plastic cell culture dishes coated with polyornithine/laminin (Merck KGaA, Darmstadt, Germany) in Dulbecco’s modified Eagle’s/F12 medium (DMEM-F12; Thermo Fisher Scientific, Waltham, MA, USA) supplemented with N2 (high transferrin, T1129, 2005, PAA Laboratories, Pasching, Austria) and containing 10 ng/mL basic fibroblast growth factor 2 (FGF2), 10 ng/mL epidermal growth factor (EGF) (both from R&D Systems, Minneapolis, MN, USA), and 1:1000 B-27 Supplement (Thermo Fisher Scientific). The cells were split every 2–3 days at a 1:2 ratio and replated at 60–80% cell confluence. Genome integrity was regularly assessed by single nucleotide polymorphism (SNP) analysis. For differentiation, the cells were plated on plastic dishes coated with Geltrex (Thermo Fisher Scientific) diluted 1:100 in DMEM-F12 medium. One day following replating, cell culture medium was changed to NGMC differentiation medium composed of DMEM-F12 medium supplemented with N2 and Neurobasal medium (both from Thermo Fisher Scientific) supplemented with B-27 1:50 mixed at a 1:1 ratio. Media change was performed every second day. After two weeks of differentiation, the cell layer was detached from the plates using StemPro Accutase (Thermo Fisher Scientific) and cells were singularized using the MACS Tissue Dissociation kit (Miltenyi Biotec, Bergisch Gladbach, Germany). Cells were cryopreserved in freezing medium, composed of KnockOut Serum Replacement (Thermo Fisher Scientific) and 10% DMSO (Merck KGaA), in liquid nitrogen (gas phase). Differentiating neural cultures were thawed by placing the cryovials in a 37 °C water bath for 3 min and subsequently resuspended in NGMC medium supplemented with Rock Inhibitor (Merck KGaA), BDNF, and GDNF (both from HiSS Diagnostics GmbH, Freiburg, Germany). Cells were directly plated at defined numbers on cell culture dishes coated with Geltrex diluted 1:50 in DMEM-F12 medium and cultured at 37 °C, 6% CO_2._ For long term differentiation, lt-NES neural cultures were maintained in NGMC medium with media changes every second day for a period of up to 10 weeks.

### 2.3. Gene Expression Analysis

Alterations of gene expression along lt-NES cells differentiation for up to 12 weeks were analyzed using whole-transcriptome microarray analysis and quantitative real-time polymerase chain reaction (qRT-PCR). For this purpose, cellular ribonucleic acid (RNA) was isolated and either directly used for microarray transcriptome analysis or reverse-transcribed into cDNA for qRT-PCR. RNA integrity was measured and validated by RNA integrity number (RIN) analysis. Only samples with RIN > 7 were used for downstream microarray analysis. mRNA expression profiles were analyzed on an Affymetrix Human Gene 2.1 ST Array microarray chip (Thermo Fisher Scientific). The Affymetrix Expression Console software was used to normalize the Affymetrix CEL file data using the Robust Multichip Average method, based on the intensity profile of the entire data set. Expression analysis was performed using Qlucore Omics Explorer software (https://qlucore.com/omics-explorer). Variance of gene expression was calculated as changes in standard deviation of the mean expression levels averaged across samples. ANOVA multigroup comparison was used for clustering analysis. Stability analysis across the differentiation time points was performed on the Affymetrix data set for several frequently used reference genes *(ACTB*, *EEF1A1*, *GAPDH*, *HPRT1*, *RPLP0*) to identify a suitable reference gene for normalization.

### 2.4. Compound Treatments

ML385 (Cayman Chemical, Ann Arbor, MI, USA) was dissolved in DMSO at a concentration of 10 mg/mL or 19.55 mM and added to the cells with media change at a concentration of 10 μM. Luperox^®^ TBHP 70% (Merck KGaA) was prediluted with ddH2O water and added to the cell media at the appropriate concentration. MG132 and Bafilomycin (both from Enzo Life Sciences, Lörrach, Germany) were dissolved in DMSO at a final concentration of 10 mM and 400 μM, respectively. For cell treatment, the compounds were administered directly to the culture media at 1:1000 dilution or up to a final concentration of 10 μM (MG132) or 400 nM (Bafilomycin). All compound treatments were performed for the indicated treatment time periods at 37 °C, 6% CO_2_.

### 2.5. Immunofluorescence Imaging

Cells were fixed in 4% paraformaldehyde for 10 min at RT, washed with DPBS and incubated with blocking solution (10% goat serum in 0.1% Triton containing DPBS) for 1h at RT. The blocking solution was replaced by antibody solution containing the primary antibody in an appropriate dilution in DPBS and the cells were incubated overnight at 4 °C. Subsequently, cells were washed with DPBS and incubated for 1 h at RT with fluorophore-coupled secondary antibody diluted 1:500 in blocking solution. Nuclei were counterstained by 2 min incubation with DAPI solution. Images were acquired on an epifluorescence microscope, observer Z1 (Carl Zeiss AG, Jena, Germany) or an INCell 2200 (GE Healthcare Life Sciences, Solingen, Germany). Fluorescence signals were detected at excitation wavelength of 555 nm (red emission), 488 nm (green), and 358 nm (blue). Primary antibodies and their working dilutions can be found in the Supplementary Information.

### 2.6. SDS-PAGE and Western Blotting

For protein sample collection, cells were cultured on 12 well plates and scraped in ice cold RIPA buffer (Merck KGaA) supplemented with 1X Halt Protease Inhibitor Cocktail (100X) (Thermo Fisher Scientific). Protein concentrations were assessed using the Pierce BCA Protein Assay kit (Thermo Fisher Scientific) and the samples were resolved on SDS-PAGE gel, 10%. Equal amounts of proteins (20 µg per lane) were transferred on a 0.4 μm pore size nitrocellulose membrane (Bio-Rad Laboratories, Hercules, CA, USA) and blocked for 1h at room temperature, rotating in 5% milk powder solution in TBS-T buffer. For LC3-II analysis, cells were collected in Sarkar Buffer (2% SDS, 10% Glycerol, 12% Urea in 10mM Tris-HCl (pH 7.4)); protein samples were run on a 15% SDS-PAGE gel and transferred on methanol-activated PVDF membrane (Bio-Rad Laboratories). Following this, blocking membranes were incubated rotating with primary antibodies at the appropriate dilution in blocking solution according to manufacturer’s instructions either overnight at 4 °C or for 2 h at room temperature. Membranes were washed 3 times in TBS-T buffer and incubated while rotating for 1 h at room temperature with secondary HRP-conjugated antibodies (Cell Signaling Technology, Danvers, MA, USA) diluted 1:1000 in 5% milk powder solution in TBS-T buffer. For chemiluminescent detection of the HRP-conjugated secondary antibody, a SuperSignal^®^ West Pico/Femto kit (Thermo Fisher Scientific) was used. The membrane was imaged in a Chemidoc XS detection system (Bio-Rad Laboratories) for analysis. Equal sample loading and transfer onto membranes was confirmed by probing GAPDH protein levels as the control (after confirmation of stability across time points). Quantification of relative protein levels was performed with ImageJ software using the Gel Analysis method.

### 2.7. ATPlite Assay

A total of 7.5–8 × 10^4^ cells were cultured per well in 96-well cell culture plates (Corning, New York, NY, USA) and differentiated for the indicated time periods. Treatment of undifferentiated lt-NES cells was performed 24h after seeding of indicated cell numbers. Cell viability following compound treatment was assessed by ATPlite assay kit (PerkinElmer, Waltham, MA, USA) according to the manufacturer’s instructions. Luminescence was measured on an EnVision plate reader (PerkinElmer) and final read-outs were obtained as relative luminescence units (RLUs). Each treatment condition was tested in at least 4 technical replicates, with biological replicates run on separate plates. Mean relative RLUs were calculated for each treatment condition and cell viability was calculated as percentage of the solvent control condition.

### 2.8. Autophagosomal Activity Assay

Autophagic vacuoles were labeled in differentiating lt-NES cells using the CYTO-ID^®^ Autophagy detection kit (Enzo Life Sciences). Differentiating lt-NES cells were cultured in 96-well black microclear bottom plates (Greiner, Kremsmünster, Austria) for the indicated differentiation/treatment time frames. Cells were pre-treated for 24 h with MG132, Bafilomycin or TBHP at the indicated concentrations and their corresponding solvent controls. After 24 h, half of the medium was removed and replaced with medium containing Cyto-ID^®^ dye up to a final dilution of 1:600, and the cells were further incubated at 37 °C, 6% CO_2_ for 90 min. Following incubation, cells were washed two times with pre-warmed PBS and fixed with 4% PFA. Images were acquired on an INCell 2200 (GE healthcare), imaging at least 8 fields per well. CYTO-ID^®^ stained autophagic vacuoles were detected with a FITC filter (excitation: 480 nm/emission: 530 nm) and a DAPI nuclear stain with a DAPI filter set (excitation: 340 nm/emission: 480 nm). CellProfiler^TM^ (www.cellprofiler.org) was used for image analysis and quantification of nuclei and autophagosome numbers. The amount of autophagosomes was normalized to the nuclei count, and autophagosomal load was presented as the autophagosomes/nuclei ratio.

### 2.9. Proteasome Activity Assay

Proteasome activity was analyzed with a fluorometric proteasome 20S assay kit (Merck KGaA), using LLVY-R110 as a fluorogenic indicator for chymotrypsin-like protease activities. Briefly, cells were cultured in 96-well black microclear bottom plates (Greiner) and differentiated for the indicated time periods. Compounds (MG132, Bafilomycin, TBHP) or solvent control was added to the cells in parallel with the fluorogenic proteasome activity indicator, and the cells were incubated at 37 °C, 6% CO_2_ for the assay duration time. Fluorescence (excitation: 485 nm/emission: 535 nm) was measured after 1 h, 2 h, 4 h, 6 h, and 8 h using an EnVision plate reader (PerkinElmer). Measurement at 1 h was used as a baseline time point, and proteasomal activity was calculated as a percentage increase in fluorescent signal relative to baseline.

### 2.10. Statistical Analysis

Student’s *t*-tests were used to assess significance between sample groups unless stated otherwise. A ratio paired *t*-test was used to compare different time points during differentiation. For comparison of multiple experimental conditions, either one-way ANOVA with post hoc Tukey (for one independent variable) or two-way ANOVA followed by Sidak’s multiple comparisons test (for interrelationship analyses) were conducted. Differences between two means with *p* < 0.05 were considered significant (* = *p* < 0.05). Error bars represent standard error of the mean ±SEM unless stated otherwise. IC20 values were calculated by performing dose–response analysis by nonlinear regression curve fit using GraphPad Prism 8.

For further details on the methods section and gene expression analysis see the Supplementary Information. For information on the primers and antibodies used see Supplementary Tables S1 and S2.

## 3. Results

### 3.1. Differentiation of Neural Stem Cell Cultures Is Accompanied by Changes in the Expression of Metabolic Pathway Genes and an Increase in Stress Resistance

**Figure 1:**
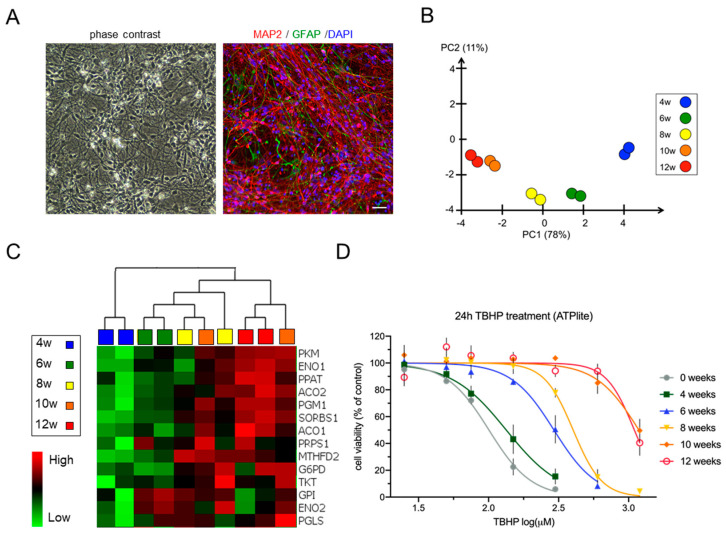
Neural differentiation of lt-NES cells is characterized by changes in metabolic pathway gene expression and increasing resistance to stress. (**A**) Phase contrast picture and a representative immunofluorescence staining of 8-week-differentiated lt-NES cells stained for MAP2 and GFAP. Scale bar, 100 µm. (**B**) Principal component analysis of differential gene expression in lt-NES cultures differentiated for 4, 6, 8, 10 and 12 weeks based on 1919 variables, showing clustering of the samples according to differentiation time. (**C**) Heatmap of glucose metabolism-associated gene expression showing upregulation of neuron-specific glycolysis genes (e.g., *PKM*, *G6PD*, *ENO1*) and pentose phosphate pathway (PPP)-associated genes (e.g., *TKT*, *PGLS*) with time of differentiation. Data were generated using an Affymetrix transcriptome microarray (*n* = 2). (**D**) Resistance to TBHP treatment increases with differentiation. Proliferating and differentiated lt-NES cells were treated with TBHP for 24 h. Toxicity was assessed by ATPlite assay at the end of the treatment phase with the indicated concentrations. Shown are measurements of the response to TBHP (after 24 h) of undifferentiated, 4, 6, 8, 10 and 12 weeks differentiated lt-NES cells, presented as percentages relative to untreated cells (equal to 100%). Data are presented as means ± SEM (*n* ≥ 3 per differentiation time point, consisting of at least 4 technical replicates each).

To study the cellular changes during neural development in vitro, we devised a long-term differentiation time frame stretching from proliferating lt-NES cells to differentiated neural cultures consisting primarily of postmitotic neurons. To standardize the various assays used in the study, cells were pre-differentiated for 2 weeks, dissociated to a single cell suspension and cryopreserved in large batches. Upon thawing and replating, these frozen cell batches give rise to highly uniform cultures containing neurons that form an extensive network of neurites. As typical for lt-NES cells, a small fraction of astrocytes emerges after prolonged differentiation. At 8 weeks of differentiation, these cultures typically contained >90% MAP2-positive neurons and around 6% GFAP-positive astrocytes (Figure 1A, as assessed on an INCell 2200 plate microscope). Previous studies have shown that lt-NES cells require at least 8 weeks of in vitro differentiation in order to generate electrophysiologically well-functioning and synaptically connected neurons [42,43]. Hence, we analyzed the cells at 4, 6, 8, 10 and 12 weeks of differentiation. Microarray-based gene expression analysis revealed almost 2000 differentially expressed genes among the neural populations differentiated between 4 and 12 weeks. Principle component analysis (PCA, Figure 1B) displayed clear sample clustering according to differentiation time. The samples from week 4 were prominently separated from week 6 and 8 samples. We thus considered the 4-week time point as the major turning point of the differentiation trajectories, given the relative distance of these data points from later time points in the PCA. Samples from weeks 10 and 12 clustered closely together, indicating a plateau phase in differentiation-associated gene expression changes (Figure 1B and Figure S1A). Lt-NES cells typically exhibit strong neurogenic potential. With increasing time of in vitro differentiation they also generate astrocytes [42,43]. In accordance, there was an increased expression of markers of glial cell differentiation with prolonged differentiation (Figure S1B), which was also reflected in an increase in immunoreactivity for the astrocytic antigen GFAP between weeks 4 and 8 (Figure S1C). The data from our microarray analysis showed expression of genes linked to essential neuronal functions such as neuro-skeleton assembly *(MAP2, NEFL)* and synaptogenesis (*SYP*), genes associated with both GABAergic and glutamatergic differentiation (e.g., *GABBR2, SLC17A6*), but also glial genes (e.g., *NFIX, S100B, AQP4*), which is in congruence with previous data about lt-NES derived neural cell types emerging upon growth factor withdrawal [42,43] (Figure S1B). Interestingly, GO term analysis performed after 4 weeks of lt-NES cell differentiation revealed the highest upregulation for genes associated with the GO term “metabolic pathways” (Figure S1D). While several other cellular pathways are changing during in vitro neural differentiation of the compound culture, our research aimed at understanding neurodevelopmental mechanisms of toxicity. Therefore we next focused on alterations in metabolism and associated cytoprotective mechanisms.

It is known that neurons rely on OXPHOS to meet their energy needs–in contrast to neural progenitor cells which utilize aerobic glycolysis instead [23]. However, glucose does not function solely as an energy source for glycolysis and OXPHOS but also fulfills important roles as a substrate for biosynthetic reactions [47]. Our microarray data indicated an upregulation of glucose metabolism-associated genes when cells were differentiated for more than 4 weeks. Interestingly, a predominant fraction of the upregulated glycolysis genes was associated with the pentose phosphate pathway (PPP) and purine biosynthesis, such as *PPAT*, *MTHFD2*, *PRPS1*, *TKT*, and *PGLS* (Figure 1C). The PPP diverges from the glycolytic pathway after the initial phosphorylation of glucose catalyzed by the glycolytic enzyme hexokinase and is a major source for NADPH regeneration in cells [48]. NADPH is used in redox reactions, and a high NADPH/NADP ratio is an important determinant for the cellular redox potential, ensuring a favorable outcome of reduction processes [49,50]. In accordance, when we measured the NADPH/NADP ratio across differentiation, we observed a continuous increase between 4 and 12 weeks of differentiation (Figure S2B).

The increased use of glucose as energy source for the maturing brain is accompanied by increased oxidation of glycolytically derived pyruvate via the TCA cycle, which generates NADH necessary for OXPHOS [47]. Indeed, our microarray data indicated an upregulation of the essential TCA enzymes aconitase 1 and 2 (*ACO1* and *ACO2*; (Figure 1C)). The switch to OXPHOS during neuronal maturation coincides with an upregulation of the mitochondrial machinery and a change in glucose metabolism [47]. In this context, we checked the expression profiles of the glycolytic enzymes Hexokinase 2 (*HK2*) and Hexokinase 1 (*HK1*), which were shown to mark metabolic transition during neuronal differentiation [24]. We observed an upregulation of *HK1* at 6 weeks of differentiation, accompanied by downregulation of *HK2* (Figure S2A). Interestingly, the 6-week time point was also marked by an upregulation in the mitochondrial biogenesis genes *PGC1-α* and *ESRRG*. These expression profiles would be compatible with a metabolic switch at around 6 weeks of lt-NES cell differentiation. 

Considering these dynamic changes, we wondered whether the cells’ response to stress would be altered across differentiation. To test this hypothesis, we treated undifferentiated and differentiated (4 to 12 weeks) lt-NES cells for 24 h with different dosages of tert-butyl hydrogen peroxide (TBHP) as a model compound for OS induction. TBHP is a potent membrane-permeable oxidant which generates free radicals and causes lipid and protein peroxidation [51]. We found that with increasing neural differentiation lt-NES cells became more resistant to TBHP stress with a 2-fold increase in the TBHP concentrations necessary to reduce the cell viability by 50% in between 4 and 6 weeks and a 4-fold increase in between 6- and 10–12-week-old cultures (Figure 1D). This finding was underpinned by the IC20 and IC50 values determined via dose–response curve analysis (Table S1). Taken together, our data indicate that the transition of undifferentiated lt-NES cells to cultures composed of neurons and glia is characterized by an increased redox state and the ability to withstand oxidative stress, which is associated with changes in the cellular metabolic profile of the cultures.

### 3.2. The KEAP1-NRF2 Pathway Is Dynamically Regulated during lt-NES Cell Differentiation

**Figure 2:**
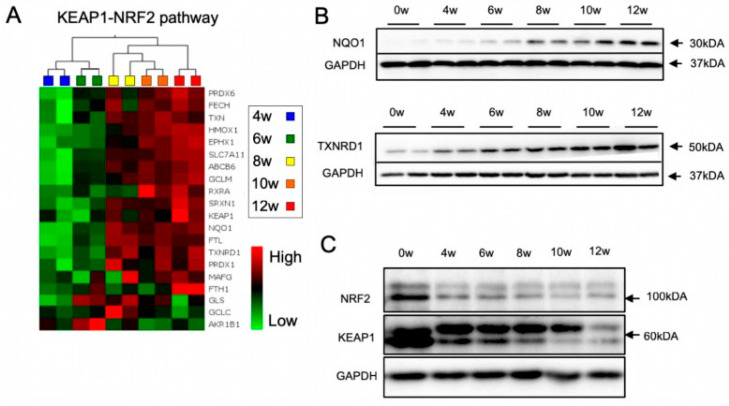
Dynamic regulation of the KEAP1-NRF2 pathway during neural differentiation. (**A**) Heatmaps showing the differential gene expression pattern of known NRF2-pathway specific genes across differentiation of lt-NES cultures. Data were generated using an Affymetrix transcriptome microarray. Expression levels (log2 scale) for each gene were averaged across samples and differences are depicted as standard deviation changes. Clustering analysis was performed by multigroup comparison (*n* = 2). (**B**,**C**) Protein expression of NRF2 pathway components was assessed by immunoblot analysis at indicated weeks (w) of lt-NES cell differentiation. (**B**) Protein levels of the NRF2 downstream targets NQO1 and TXNRD1 increase during neural differentiation. (**C**) NRF2/KEAP1 protein levels decrease during neural differentiation.

Given the crucial role of cellular redox balance for cell survival and differentiation, the observed changes in cellular metabolism and the increased stress resistance prompted us to assess changes in the antioxidant response signaling during lt-NES differentiation. Considering its eminent role in orchestrating cytoprotective mechanisms, we focused on the NRF2 pathway as a major regulator of the antioxidant response in different tissues, including the brain. NRF2 downstream genes encode for a diverse set of adaptive programs, which protect against environmental and, in particular, oxidative stress. Indeed, our microarray data showed a prominent increase in the expression of glutamate-cysteine ligase modifier (*GCLM*), heme oxygenase 1 (*HMOX1*), NAD(P)H quinone acceptor oxidoreductase 1 (*NQO1*), sulfiredoxin1 (*SRXN1*), thioredoxin reductase 1 (*TXNRD1*) and several other NRF2 target genes after 6 weeks, reaching a plateau phase after 8 weeks of differentiation (Figure 2A). Western blot analysis confirmed upregulation *NQO1* and *TXNRD1* (Figure 2B). Both proteins are important players in the cells’ defense mechanism against ROS. NQO1 maintains cellular redox homeostasis by mediating the two-electron reduction of quinones to hydroquinones [52], while TXNRD1 is the main enzyme propelling the thioredoxin 1 system, which reduces oxidized cysteine residues and peroxiredoxins [53]. Our data thus indicate that the NRF2 pathway is upregulated with neural differentiation of lt-NES cells, in parallel with an increase in the cells’ potency to respond to stress.

NRF2 mediates the induction of hundreds of downstream target genes, often in interplay with other transcription factors [54,55,56]. For example, the *HMOX1* gene is regulated by at least four other transcription factors in addition to NRF2 [57]. We became interested in assessing the expression of NRF2 downstream target genes upon induction of oxidative stress by TBHP and the question whether they are exclusively targeted by NRF2. OS induction by TBHP showed upregulation of the four tested downstream target genes *NQO1*, *TXNRD1*, *SRXN1*, and *HMOX1*. Specificity for NRF2-dependent regulation was checked by concomitant application of ML385, a pharmacological inhibitor of NRF2. While TBHP induced upregulation of all four target genes, co-treatment with ML385 clearly dampened this response for *NQO1* and *TXNRD1* (Figure S3A), indicating that both genes are specifically regulated by NRF2 and can be used as surrogate markers for NRF2 activity in differentiated lt-NES cultures. We also assessed the protein levels of the two main components of the NRF2 pathway—NRF2 and its negative regulator KEAP1—and found a pronounced decrease in both proteins upon differentiation (Figure 2C). This was an unexpected finding as the changes in NRF2 protein levels did not match the upregulation of NRF2 downstream targets. Furthermore, the microarray data suggested that the expression of *NRF2* increases at later stages of differentiation, while *KEAP1* mRNA remains rather stable (Figure S3B). Together, these findings suggest that the two proteins are regulated by post-transcriptional mechanisms, e.g., active turnover by the proteostatic machinery. They also indicate that the NRF2-KEAP1 pathway is dynamically regulated throughout differentiation.

### 3.3. The NRF2-KEAP1 Signaling Pathway Contributes to Increasing Resistance to Oxidative Stress with Neural Differentiation

**Figure 3:**
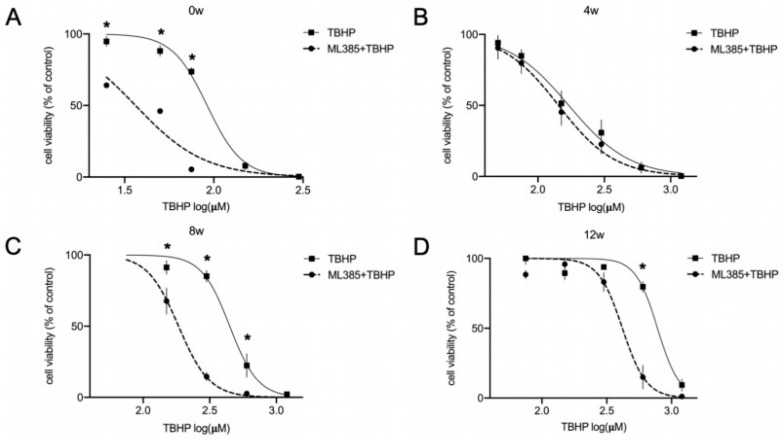
NRF2-KEAP1 signaling contributes to increased resistance to oxidative stress during differentiation of lt-NES cells. (**A**–**D**) Contribution of the NRF2 pathway to the OS response in differentiating lt-NES cultures was assessed using the NRF2 inhibitor ML385. Cell viability in response to oxidative stress was assessed in undifferentiated lt-NES cells (**A**) and lt-NES cells differentiated for 4 (**B**), 8 (**C**) and 12 (**D**) weeks after 24 h pre-treatment with ML385 followed by 24 h tert-butyl hydroperoxide (TBHP) treatment. Toxicity was assessed by ATPlite assay at the end of the treatment period with the indicated concentrations. Data are presented as means ± SEM (*n* ≥ 3, consisting of at least 4 technical replicates each). Significant difference between TBHP treatments with or without ML385 was confirmed by two-way ANOVA (Interaction *p* values: 0 w *p* < 0.0001; 4 w *p* = 0.9990; 8 w *p* < 0.0001; 12 w *p* < 0.0001) followed by Sidak’s multiple comparisons test (* = *p* < 0.05).

The upregulation of NRF2 pathway downstream targets, including *NQO1* and *TXNRD1* together with the clear indication for higher redox potential with neural maturation prompted us to investigate whether the NRF2 pathway is contributing to the observed increasing resistance to stress with differentiation. For this purpose, we inhibited NRF2 using the pharmacological inhibitor ML385, co-treated the cells with TBHP, and analyzed cell viability by ATPlite assay. For this analysis, we focused on undifferentiated lt-NES cells, immature (4 weeks), and mature (8 and 12 weeks) neural cultures. In accordance with the dynamic changes in NRF2 pathway activity during differentiation, we observed that the impact of this pathway on cell survival varies greatly between the different time points (Figure 3A–D). While TBHP exposure in the presence of the inhibitor clearly affected the viability of proliferative (Figure 3A) and 8 to 12-week-differentiated lt-NES cells (Figure 3C,D), the NRF2 pathway appeared not to contribute to the stress response in 4-week-old cultures (Figure 3B).

Differentiated lt-NES cells were not affected by NRF2 inhibition in the absence of oxidative stress induction (data not shown). However, proliferative lt-NES were sensitive to the reduction in NRF2 protein levels by ML385 (Figure S4). To account for the high turnover of NRF2 protein in proliferative lt-NES cells, proteasomal inhibition with MG132 was used to validate the decrease in total NRF2 load following ML385 treatment (Figure S4A). In parallel to this we observed a marked decrease in cell viability, assessed by ATPlite, upon NRF2 inhibition (Figure S4B). Together, these data indicate that the NRF2 pathway is crucial for the survival of proliferating lt-NES cells and contributes to the increasing resistance to stress with neural maturation as shown in Figure 1D.

### 3.4. The Autophagy Pathway Is Upregulated during Neural Differentiation

In order to shed light upon the mechanisms underlying the dynamic modulation of the NRF2 pathway across differentiation and the increasing resistance to stress, we next focused on molecular pathways that are tightly linked to both stress response and NRF2 signaling and that are suspected to show alterations across differentiation as well. One obvious candidate pathway is autophagy, i.e., hydrolytic degradation of superfluous and potentially toxic cellular components within the lysosomal compartment. Autophagy is strongly upregulated under various stress conditions (such as proteotoxic, oxidative and nutrient deprivation-related stress [58]) in order to maintain cellular homeostasis. The NRF2 inhibitor KEAP1 is a known target of autophagic degradation, and the autophagic protein p62 acts as an adaptor protein in this process [37]. As autophagy dynamics have also repeatedly been linked to development and differentiation events, we first sought to assess potential changes in autophagic components along differentiation.

**Figure 4:**
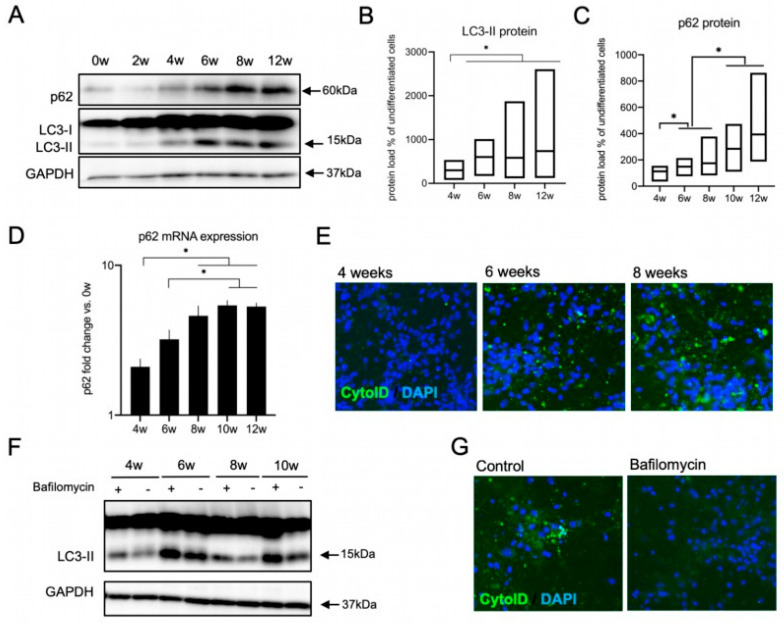
Upregulation of the autophagic machinery during neural differentiation. (**A**–**E**) Increase in the autophagic components with ongoing neural differentiation of lt-NES cultures. (**A**) Representative Western blot analysis of LC3-II and p62 protein expression along differentiation. (**B**,**C**) Quantification of Western blot data of LC3-II (**B**) and p62 (**C**) protein levels in lt-NES cells differentiated for up to 12 weeks. Data are presented as percentage of LC3-II levels in undifferentiated cells ±SEM (floating bar represents the range of the SEM value; the mean is marked within each bar). All samples were normalized to GAPDH as a loading control. Statistical significance was assessed by ratio paired *t*-tests (* = *p* < 0.05; *n* = 8). (**D**) Expression of the adaptor protein p62 increases along lt-NES neural differentiation. Data are normalized to *GAPDH* mRNA levels (*n* = 6 per differentiation time point). Significant difference among means was confirmed by one-way ANOVA (*p* = 0.0001) followed by Bonferroni’s multiple comparison tests (* = *p* < 0.05). (**E**) Representative images of 4-, 6-, and 8-week-differentiated lt-NES cells stained with a CYTO-ID^®^ Autophagy detection kit. Nuclei were counterstained with DAPI. (**F**,**G**) Autophagic flux is active in differentiating lt-NES cells. (**F**) Autophagic flux assessment across lt-NES cells differentiated for up to 10 weeks, showing active autophagic flux at all differentiation stages. Cells were treated with Bafilomycin (400 nM) for 4 h. (**G**) Representative images of 10-week-differentiated lt-NES cells treated with Bafilomycin and stained with CYTO-ID^®^ Autophagy detection kit. Nuclei were counterstained with DAPI.

We focused on LC3-II and p62 as widely used markers for the assessment of the autophagic state in cells. LC3-II is formed after the proteolytic processing of full-length LC3 protein (‘LC3-I’) and its conjugation with phosphatidyl-ethanolamine (PE). By this modification, LC3-II is localized to isolation membranes and mature autophagosomes, and its relative amount directly reflects the number of autophagosomes. p62 (also known as Sequestosome 1, encoded by the *SQSTM1* gene) is an important autophagic receptor protein that links cellular substrates to LC3 on the growing autophagosome [59]. P62 can be used to monitor autophagic activity as it mediates the selective degradation of cargo trapped within or bound to autophagosomes, including its own [60]. When we looked at the autophagic marker LC3-II, we found a prominent upregulation of the LC3-II protein levels starting at 4 weeks of differentiation with subsequent plateauing at 6–12 weeks (Figure 4A,B). P62 protein was also upregulated with an incremental increase with differentiation on both protein (Figure 4A,C) and mRNA level (Figure 4D). Next we wanted to investigate if the increase in the autophagic components LC3-II and p62 is associated with an upregulation in autophagic activity with differentiation. For this purpose, we used CytoID dye, which detects specifically active autophagosomes and autolysosomes. Indeed, we found an upregulation in the staining of active autophagic sites in lt-NES cells differentiated for 6 and 8 weeks (Figure 4E). We also confirmed activity of the lysosome-dependent degradation along differentiation using Bafilomycin, a V-ATPase inhibitor, which blocks the acidification and fusion of the autophagosomes with the lysosome, thereby resulting in accumulation of autophagosomes in cells. The autophagic flux level is represented by the difference in the amount of LC3-II (as a proxy for autophagosomal structures) between samples with or without Bafilomycin treatment. Indeed, blocking autophagosomal flux by Bafilomycin resulted in an accumulation of LC3-II protein (Figure 4F) in differentiating lt-NES cells and ablation of CytoID signal intensity (Figure 4G), confirming that autophagic flux is active. In addition, these data exclude the possibility that the increase in LC3-II and p62 is due to stalled autophagosomal turnover. Taken together, the increase in p62 transcript together with the upregulation of acidified autophagosomes indicates that there is a clear upregulation of both components of the autophagic machinery and autophagic flux after around 6 weeks of lt-NES differentiation.

### 3.5. Autophagic Flux Does Not Contribute to the Acute Oxidative Stress Response in Differentiated lt-NES Cells

**Figure 5:**
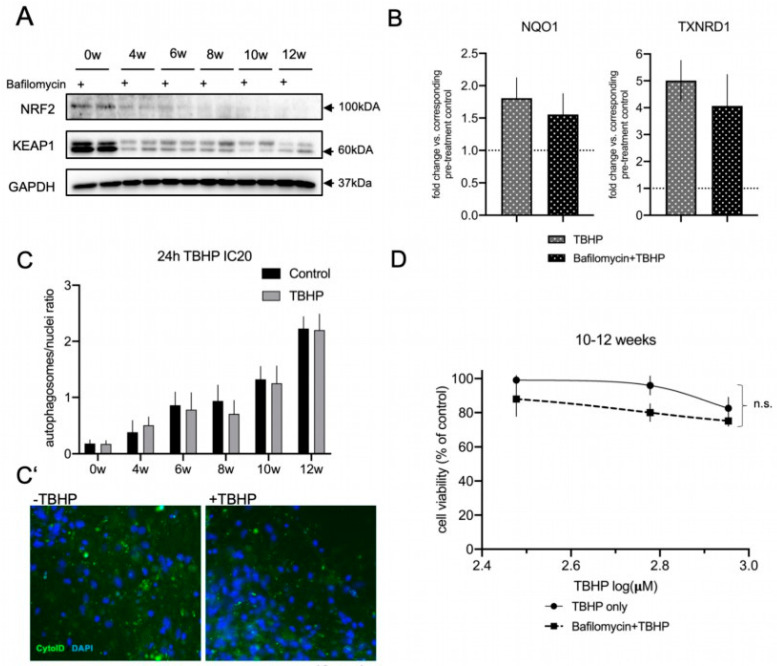
Autophagic flux does not contribute to the acute oxidative stress response in differentiating lt-NES cells. (**A**,**B**) Crosstalk between autophagy and KEAP1-NRF2 pathway regulation. (**A**) Western blot analysis of NRF2 and KEAP1 protein levels across lt-NES differentiation upon autophagic flux inhibition (by Bafilomycin) for 17 h. (**B**) NRF2 pathway downstream target gene expression under OS induction upon autophagic flux inhibition. lt-NES cells were differentiated for 10 weeks and pre-treated with Bafilomycin for 17 h, followed by addition of TBHP in the presence of Bafilomycin for 8 h. Cells were treated with TBHP only for 8 h as a control treatment. Expression of the NRF2 downstream target genes *NQO1* and *TXNRD1* was analyzed by qRT-PCR in order to analyze the effects on NRF2 pathway activation upon oxidative stress (TBHP) under basal conditions or during autophagy inhibition. Results are presented in comparison to the corresponding pre-treatment condition (Bafilomycin or pre-treatment control; equal to 1). Data are normalized to GAPDH mRNA levels and presented as means ± SEM (*n* = 4 per treatment condition). Significant difference between means for each treatment condition was determined by Student’s t test (*NQO1 p* = 0.5991; *TXNRD1 p* = 0.5257). (**C**) Autophagic flux response to 24 h TBHP IC20 treatment in lt-NES and lt-NES cultures differentiated up to 12 weeks quantified by CYTO-ID^®^ staining shows that TBHP treatment does not induce upregulation of the autophagosomal flux. The autophagosomal load was assessed by calculating the ratio of autophagosomes to nuclei (*n* = 3, consisting of at least 3 technical replicates per treatment condition each). (**C’**) Representative CYTO-ID^®^ staining images of 12-week-differentiated lt-NES cultures show no increase in autophagosomal load upon TBHP treatment. Nuclei were counterstained with DAPI. (**D**) ATPlite assay of pooled 10–12-week-differentiated lt-NES cells treated with TBHP with or without Bafilomycin. Cells were pre-treated with Bafilomycin for 17 h followed by a 24 h TBHP treatment. Cell viability was assessed at the end of the treatment by ATPlite assay. Results are presented as percentages relative to untreated cells (equal to 100%) ±SEM (*n* = 5, consisting of at least 3 technical replicates per treatment condition). Significance was assessed by two-way ANOVA (*p* = 0.89).

Since p62 is a known regulator of NRF2 by mediating the autophagic degradation of KEAP1, we were wondering whether the increase in autophagosomal machinery is affecting the NRF2 pathway in differentiated lt-NES cells and if it contributes to the dynamic stress response along differentiation in an NRF2-dependent or independent manner. To tackle this question, we blocked autophagy for a continuous time frame and analyzed the effect on NRF2 and KEAP1 proteins. Our assumption was that blocking autophagic flux would result in KEAP1 accumulation; however, we saw no effect on either protein in our experimental setup (Figure 5A). Next, we assessed the NRF2-related response to acute stress in the presence of autophagosomal inhibition. To that end, we first confirmed that long-term treatment (up to 24 h) with Bafilomycin results in LC3-II protein accumulation (Figure S5). To ensure efficient inhibition, we started with a pretreatment with Bafilomycin and then induced oxidative stress with TBHP alone or in combination with Bafilomycin. We saw that in accordance with the absence of alterations in the NRF2 and KEAP1 protein levels, treatment with TBHP induced the expression of the NRF2 downstream targets *NQO1* and *TXNRD1* also in the presence of Bafilomycin, indicating that the NRF2 response is active also in the absence of autophagic flux in differentiated lt-NES cells (10 weeks; Figure 5B). This result suggests that NRF2 regulation under acute stress conditions is independent of the state of the autophagic flux. However, this does not exclude the possibility that the increase in the autophagic machinery with differentiation is playing an important role in the resistance to stress in an NRF2-independent manner. We next set out to assess the actual contribution of autophagy to the stress response in differentiated lt-NES cells, since autophagy is known to be induced by multiple stress pathways to serve as an adaptive mechanism for maintenance of bioenergetic homeostasis, recycling of misfolded and aggregate-prone proteins, and removal of aged, uncoupled or permeabilized mitochondria [61]. We used doses of TBHP according to calculated IC20 concentrations for the respective differentiation time points and treated proliferative and differentiating lt-NES cells for 24 h, followed by CytoID autophagosomal stain. We did not see any TBHP-induced upregulation of the acidified autophagosomal load at any differentiation time point, indicating that there is no response of the autophagic flux upon 24 h OS induction in lt-NES cultures (Figure 5C). We also studied whether blocking autophagy in the most differentiated cultures (10–12-weeks-old) would impact cell viability. We pre-blocked autophagy using Bafilomycin for 17 h and afterwards treated the cells with low toxic doses of TBHP (based on the estimated IC20 concentrations for this differentiation time points). Treatment with TBHP alone was used for the generation of a control response curve. In accordance with the absence of a response to acute stress (Figure 5C), we found no significant differences between the control and Bafilomycin/TBHP treatment dose–response curves (Figure 5D), indicating that blocking autophagy does not increase the susceptibility to oxidative stress in differentiated lt-NES cells.

Taken together, these findings indicate that autophagy does not regulate the NRF2 pathway under acute OS conditions and that autophagic flux activation is not a frontline response to acute stress conditions in differentiated lt-NES cultures.

### 3.6. The Proteasome Is a Major Contributor to the Acute Oxidative Stress Response in Differentiating lt-NES Cells and Regulates the NRF2-KEAP1 Pathway Response

**Figure 6:**
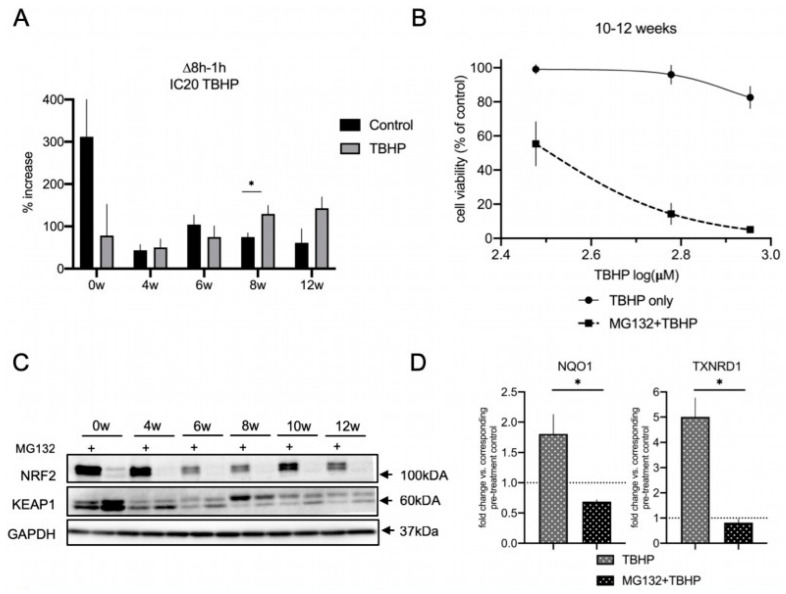
The proteasome is a major contributor to the acute oxidative stress response in differentiating lt-NES cells and regulates NRF2 pathway activation. (**A**) Measurement of the 20S proteasome activity in proliferating and differentiated lt-NES cells. The proteasomal substrate dye LLVY-R110 was added to the cells together with IC20 TBHP concentrations, and fluorescence was measured after 1 h and 8 h. Proteasomal activity is presented as percentage increase in the fluorescence signal between 8 h and the baseline value at 1 h (∆t-t1h). Data are presented as means ± SEM (*n* = 4 per treatment and differentiation time point, * *p* < 0.05, Student’s *t*-test). (**B**) ATPlite assay of lt-NES cells differentiated for 10 and 12 weeks treated with TBHP with or without MG132 pre-treatment, showing that proteasomal impairment leads to increased susceptibility to acute oxidative stress. Cells were pre-treated with MG132 followed by a 24 h TBHP treatment. Cell viability was assessed at the end of the treatment by ATPlite assay. Results are presented as percentages relative to untreated cells (equal to 100%). Data are presented as means ± SEM (*n* = 5, consisting of at least 3 technical replicates per treatment condition; data from the control curve are the same as shown in Figure 5D). Significant difference between dose–response curves for TBHP treatment with or without MG132 was confirmed by two-way ANOVA (*p* < 0.0001). (**C**) Western blot analysis of NRF2 and KEAP1 protein levels across lt-NES differentiation upon proteasomal inhibition (by MG132) for 17 h. (**D**) NRF2 pathway downstream target gene expression under OS induction upon inhibition of the proteasome (MG132). lt-NES cells were differentiated for 10 weeks, pre-treated with MG132 for 17 h and subsequently treated with TBHP for 8 h. Cells were treated with TBHP only for 8 h as a control treatment. Expression of the NRF2 downstream target genes *NQO1* and *TXNRD1* was analyzed by qRT-PCR. Results are presented in comparison to MG132 pre-treatment control (equal to 1). Data are normalized to *GAPDH* mRNA levels and presented as means ± SEM (*n* = 4 per treatment condition). Statistically significant difference between means was assessed by unpaired Student’s *t*-test for *NQO1* (*p* = 0.0128) and *TXNRD1* (*p* = 0.0016) gene expression (* *p* < 0.05).

Based on our finding that autophagy is not contributing to the acute response to oxidative stress in differentiating lt-NES cells, we decided to examine the contribution of the second major proteostasis machinery, the proteasome, to the stress response. Beyond participating in the clearance of ubiquitinylated proteins as part of its physiological functions, the proteasome is also an important part of the defense system against oxidative stress. This is particularly true for mounting a cellular regulatory response to stress and the degradation of oxidized proteins with the 20S proteasome core particle playing a central role in this process [62]. We assessed the 20S catalytic core subunit β5, which is characterized by chymotrypsin activity and plays a rate-limiting role for proteasome assembly and activity [63,64]. Interestingly, 20S β5 subunit protein levels did not increase with differentiation (Figure S6A). qRT-PCR analysis also showed no significant changes in the transcript levels across differentiation (Figure S6B). To obtain more comprehensive insight into proteasomal performance during differentiation we turned to a functional activity assay. We used the proteasomal substrate dye LLVY-R110, which emits fluorescence signal upon proteasomal degradation, thereby enabling direct quantification of proteasomal activity. We measured the percentage of the increase in basal and acute oxidative stress-induced proteasomal activity using low toxic IC20 doses of TBHP for 8 h (corresponding to the respective differentiation time point; Figure 6A). Specificity of the assay for proteasomal degradation activity was confirmed by proteasomal inhibition with MG132 treatment, which resulted in ablation of the fluorescence signal (Figure S7). In accordance with the rather uniform expression of the proteasomal β5 subunit, we did not see any upregulation of basal proteasome activity with differentiation. In fact, basal proteasomal activity in proliferative lt-NES cells was around six to seven times higher than in differentiated lt-NES-derived cultures. However, upon oxidative stress challenge we detected changes in these activation states, with proteasomal degradation being largely ablated in undifferentiated cultures and upregulated at late stages of differentiation. Young differentiating cultures (4 and 6 weeks) did not show upregulation upon oxidative stress challenge, while mature cultures (8 and 12 weeks) showed on average higher proteasomal activation in comparison to basal conditions (Figure 6A). These observations prompted us to explore whether proteasomal degradation is an important component of the response to acute cell stress in fully differentiated lt-NES cells. To address this question, we measured cell viability upon oxidative stress challenge following proteasomal inhibition with MG132 in 10–12-week-differentiated cultures. Specifically, we performed a 17 h pre-treatment with MG132 and subsequently replaced it with TBHP for 24 h. We used low toxic doses, based on the estimated IC20 concentration under standard culture conditions, which resulted in a flat response curve as expected (Figure 6B). However, when we applied the same doses following MG132 pre-treatment we detected a dramatic decrease in cell viability at concentrations which result in minimal toxicity under control conditions. These results indicate that impairment of proteasomal function disrupts the ability of mature neural cultures to cope with stress.

Interestingly, we also observed that proteasomal inhibition for 17 h results in an upregulation of autophagic flux (based on the CYTO-ID assay; Figure S8A,A′) and p62 (Figure S8B). However, looking at the strong decrease in cell viability upon proteasome inhibition (Figure 6B), this phenomenon was apparently not able to mitigate the stress response.

Lastly, we sought to investigate whether NRF2 is involved in the observed contribution of proteasomal activity to the acute stress resistance in mature lt-NES cell cultures. The NRF2 protein itself is a known target of proteasomal degradation [20], and accordingly we saw that MG132 treatment results in accumulation of NRF2 protein, confirming that NRF2 is actively turned over by the proteasome in lt-NES cells and their differentiated derivatives (Figure 6C). Interestingly, we found that KEAP1 protein levels are reproducibly downregulated upon proteasomal inhibition both in precursor and differentiated cells (Figure 6C and Figure S9).

The abundance of NRF2 protein implies that the pathway is active and able to efficiently respond to stress, so we sought to investigate if this is the case under TBHP-induced oxidative stress conditions in lt-NES cultures. To this aim, we investigated the state of the NRF2 pathway under MG132 treatment in the absence and presence of oxidative stress. Contrary to our assumption, upon proteasomal inhibition there was ablation of the NRF2 downstream target gene response following TBHP treatment, as shown by assessing *NQO1* and *TXNRD1* expression levels (Figure 6D). These results argue for proteasomal degradation to be a frontline response to acute stress in mature lt-NES cultures. The differential response of NRF2 and KEAP1 levels to MG132 further suggests that proteasomal degradation plays a key role in regulating the acute NRF2 cytoprotective response to oxidative stress by maintaining proper ratios of NRF2 and KEAP1 protein levels. We also confirmed the observed effects of proteasomal inhibition in younger (6 weeks differentiated) neural cultures (Figure S10A). As seen in 10-week-differentiated cells (Figure 5B and Figure 6D), autophagic flux ablation, again, did not affect the TBHP stress-induced NRF2 response (Figure S10A). Cell viability data for treatment with IC20 doses of TBHP also showed that proteasomal impairment, but not autophagic flux inhibition results in a drastic increase in the susceptibility to OS (Figure S10B). These data suggest that proteasomal regulation of the oxidative stress response is a general feature in differentiated lt-NES cell cultures.

## 4. Discussion

### 4.1. A Model of Dynamic Changes in Stress Response Patterns during Neural Differentiation

In vitro neural differentiation of hPSCs has been shown to follow the rules of in vivo development [65], and hiPSCs use the same transcriptional networks to generate neuroepithelial cells and functionally distinct neural subtypes over the same developmental time course as human embryonal stem cells (hESCs) [66]. This reinforces the use of hPSC technology to study and model the otherwise inaccessible mechanisms underlying human neural development in vitro. Neural differentiation of hPSCs transits through defined stages, corresponding to distinct NPC populations found in distinct stages of embryonic and fetal developmental [67,68]. Rosette-type NPCs resemble early precursor cells populating the neural tube during neurulation. They express early neuroectodermal markers, are capable of extensive self-renewal, and have a broad differentiation potential along CNS and PNS lineages [69]. Along the same line, the lt-NES cells used in this study do not represent mere in vitro proxies of undefined neural cell populations but closely resemble cells that can be isolated from the embryonic human brain. Specifically, lt-NES cells have been shown to exhibit striking similarity to neuroepithelial stem cells derived and expanded from early human embryos (week 5–7, Carnegie stage 15–17) [70]. These observations underline the value of lt-NES cells as system to experimentally dissect and model aspects of early human CNS development.

**Figure 7:**
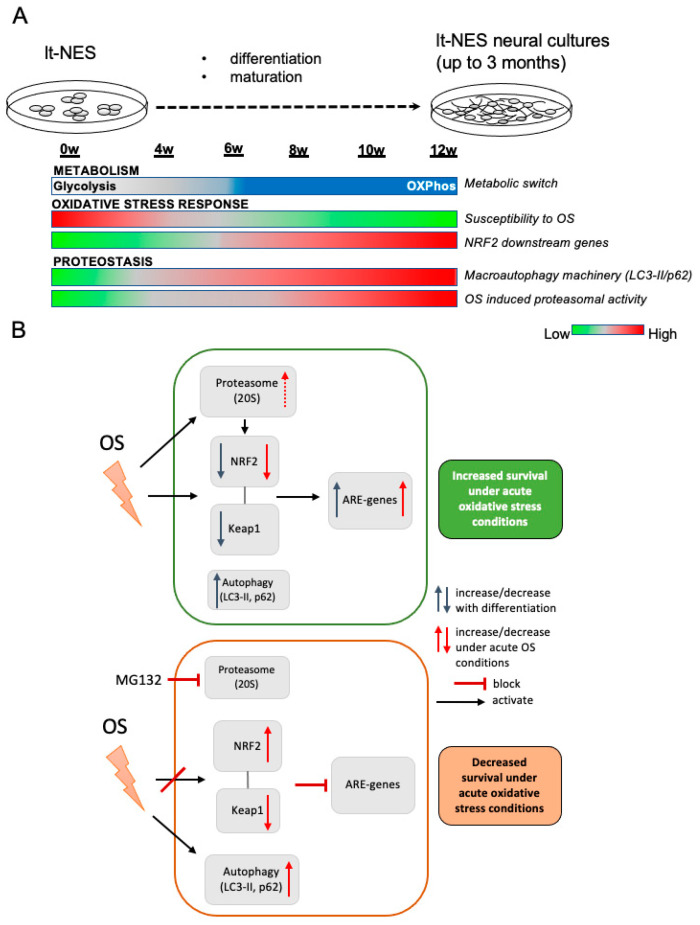
The oxidative stress response during neural differentiation is tightly coupled to developmental changes in proteostasis and NRF2-KEAP1 pathway modulation. (**A**) Schematic representation of alterations in OS-associated pathways across neural differentiation. (**B**) Schematic representation of the interaction between proteostasis (autophagy and proteasome) and the NRF2-KEAP1 pathway under physiological and acute OS conditions. Top panel: OS response control under functional proteostasis conditions. Bottom panel: OS response under dysfunctional proteostasis induced by proteasome inhibition.

Here, we used this human in vitro model to assess the dynamics of cell metabolism, autophagic and proteasomal degradation, together with changes in the cells’ redox potential and NRF2-dependent stress response pathways during early neural differentiation (Figure 7). In particular, we looked at how these processes converge in the way neural cultures respond to stress and describe alterations, which are an integral part of the differentiation process (Figure 7A). We see an upregulation of the NRF2 pathway downstream target activation, accompanied by dynamic changes in the NRF2 and KEAP1 protein and mRNA levels, with a decrease in NRF2 and KEAP1 proteins. At the same time, proteostatic processes mediated by autophagy are also dynamically changed with an upregulation of p62 and autophagic flux. NRF2 protein is constantly being subjected to proteasomal degradation and a ratio between NRF2 and its regulator KEAP1 is thus maintained. Changes in cell metabolism are associated with upregulation of cellular ROS levels [71] which leads to upregulation of redox pathways such as NRF2 in order to maintain cellular homeostasis. In accordance with an adaptive response to the increasing levels of ROS, the cells increase their capability to cope with stress (Figure 7B; upper panel), which we confirmed by observing an increasing resistance to acute TBHP toxicity. Already at 6 weeks we observed a higher potential to respond to stress, which is significantly increasing at 8 weeks and onwards. This increasing resilience towards cellular stress may result from progressing neuronal differentiation and could also be due to the emergence of astrocytic cells at later stages of lt-NES differentiation. As in physiological neurodevelopment, the increasing cellular complexity with the appearance of astrocytic cells may exert cytoprotective effects on the neuronal compartment. 

When we examined how proteostasis is affecting the cells’ resilience to stress and its interaction with the NRF2 antioxidant response, we found that proteasomal degradation is crucial for maintenance of the protein levels of NRF2 and KEAP1 allowing the pathway to function upon stress challenge, even in the absence of autophagic degradation. When proteasomal degradation is impaired, autophagic flux is overactivated as a compensatory mechanism (Figure 7B, lower panel). In parallel to this phenomenon, we observe a decrease in KEAP1 protein and accumulation of NRF2. The dysbalanced NRF2-to-KEAP1 ratio is accompanied by pathway deactivation and decreased ability of the cultures to respond to stress. Importantly, as our cellular system gives rise to neural progeny of both neuronal and glial cell types, but not of microglial or endothelial cells, it will be interesting to investigate in the future how complementing our compound neural culture with these cell types affects the resilience and/or vulnerability of the mixed population under various stress conditions.

### 4.2. Changes in Metabolism during Neural Differentiation Are Associated with Increased Resistance to Stress

Hawkins and colleagues showed that during reprogramming of human dermal fibroblasts to iPSCs there is an increase in mitochondrial respiration as well as channeling of glucose through the pentose phosphate pathway (PPP) in order to manage increased nucleotide synthesis demands. In their study, the peaks in OXPHOS and PPP correlate with maximal NRF2 activity [72]. Our data could point to a similar phenomenon during neural differentiation of lt-NES cells, where we observed increased expression of pentose phosphate pathway (PPP)-associated genes with prolonged differentiation. This could suggest that NRF2 activity is changing with the metabolic alterations accompanying the differentiation processes. In the developing brain, metabolism via the PPP is particularly important to provide building blocks for biosynthetic processes [47]. In human models, differentiation in SH-SY5Y cells was also shown to result in upregulation of the PPP [73]. The increase in NADPH/NADP ratio with differentiation supports the notion that the metabolic changes are associated with increased activity of the PPP. In addition, OXPHOS activity is known to lead to an increase in ROS levels as a byproduct of mitochondrial respiration [74], and antioxidant defense in neurons is crucial to protect mitochondria from this physiological phenomenon [75]. Recently, a detailed study of metabolic reprogramming during neural differentiation up to 3 weeks of iPS-derived rosette-type NPCs was reported [24]. The authors observed an increase in the mitochondrial biogenesis associated genes *PGC1α* and *ESSRG* and the neuronal glycolysis gene Hexokinase 1 (*HK1*) in cultures differentiated for 3 weeks in comparison to the precursor stage. In accordance with their findings, we also see evidence for an upregulation of these genes with lt-NES cell differentiation. Alongside these changes, we detect upregulation of cellular defense pathways and an increase in the overall ability of the cells to withstand stress. Our findings delineate 6 weeks of differentiation as the time point at which most dynamic changes take place in our lt-NES cell model system. After this time point, we also observed a pronounced and continued increase in protein levels of the autophagic adaptor p62. p62 was found to be an essential player in neural differentiation by regulating Akt pathway activation [76], and a recent study using iPSC-derived neurons and neuronal precursor cells from patients carrying a p62 nonsense mutation also showed that p62 is essential for neuronal differentiation by controlling the metabolic shift from aerobic glycolysis to OXPHOS [77]. P62 is also a known regulator of the NRF2 pathway. The adaptor protein directly interacts with the NRF2 regulator KEAP1, promoting its autophagic clearance, which results in subsequent upregulation of the NRF2 response [38,78,79]. It is therefore conceivable that the decrease in KEAP1 protein levels we observe upon differentiation is an inherent part of the neurodevelopmental process. This decrease would promote the activation of the NRF2 pathway which we observe in mature neural cultures. At the same time there is evidence for a positive feedback loop mechanism between p62 and NRF2 [80], which would explain the increase in p62 mRNA levels especially in more differentiated cultures.

### 4.3. Dynamic Activation of NRF2 Pathway along lt-NES Cell Differentiation

A recent study by Bell et al. reported that NRF2 is transcriptionally silenced when embryonic rat cortical neurons are cultured for 9 days in vitro (DIV9) [21]. This event is believed to be necessary only during early neural differentiation stages in order to let key developmental pathways to take place and was observed in a specific developmental window between very immature (DIV2) and more developed cultures (DIV14). Interestingly, in our differentiation paradigm we see that at 4 weeks of lt-NES cell differentiation NRF2 pathway activity is lower in comparison to later stages and that it does not contribute to the resistance to stress. It is conceivable that 4 weeks is still a time period of neuronal differentiation which is marked by morphological changes and growth as shown by our microarray data. Therefore, it is possible that pathway silencing occurs at this stage to allow developmental processes to take place. Moreover, we generally see lower NRF2 protein levels in differentiated lt-NES as compared to proliferative cells, which could be explained by the NRF2 promoter silencing events in neurons described by Bell et al. [21]. Interestingly, upon MG132 proteasomal inhibition, we see an accumulation of NRF2 protein across all differentiation stages, indicating that NRF2 protein is available in cells but actively turned over by the proteasome. Further studies will be necessary to unravel the complex interplay between promoter silencing events and proteostatic dynamics. Moreover, it is important to note that Bell et al. showed that NRF2 silencing is specific for neurons but not glia cells. However, while lt-NES cells mostly give rise to neurons, they also generate a minor fraction of astrocytes, which can already be observed at 4 weeks of differentiation [42,43]. Indeed, we observed an expanding glia population and upregulation of gliogenic switch markers such as *NFIX* and *AQP4* [81] at later stages of maturation.

The emergence of glial cells in differentiating lt-NES cell cultures provides both advantages and limitations. On the one hand, the glial compartment is an indispensable component of the developing human nervous system and promotes neuronal maturation [82,83]. In fact, many of the phenotypes observed in our study as well as mechanisms, facilitating these effects, may not be observable in purified cell populations, due to reduced fitness and viability of, e.g., pure neuronal cultures. On the other hand, the presence of glial cells instills non-cell-autonomous effects and can thus limit a clear allocation of observed phenomena to a distinct neural subtype. Despite this limitation, neural stem cell populations such as lt-NES cells, which can also be derived from fetal human brain [70], might be particularly suitable for studying metabolic changes and stress response mechanisms at a population level because they mimic the emerging cellular heterogeneity observed during CNS development. Further studies involving single cell analyses might be able to tackle the challenge of cellular heterogeneity and thus enable combined analysis of population effects and cellular subtype-specific changes.

### 4.4. Modeling of Autophagic Degradation during Neurodevelopment and Its Role in the Acute Response to Stress in Differentiating lt-NES Cells

Human PSC-derived neurons are a compelling tool to study mechanisms such as autophagy considering the importance of this process for cellular homeostasis and disease [84]. To our knowledge, this study provides the first detailed timeline of dynamic changes in autophagy along the differentiation of neural stem cells, generated from human PSC. We observed an upregulation of the autophagic machinery with differentiation and a pronounced difference in the protein homeostasis processes between undifferentiated lt-NES cells and their differentiated progeny. Indeed, autophagy induction is associated with facilitation of cell differentiation [85,86], while lower autophagy activity is necessary under proliferative conditions [87]. Autophagy is a central prerequisite for the neurodevelopmental process, presumably due to its role in providing molecular building blocks from recycled cellular constituents. In our experimental paradigm, we see an upregulation in both the autophagic machinery and autophagic flux across neural differentiation. In a similar manner, a recent study showed that nerve growth factor (NGF) induced neuronal differentiation results in upregulation of LC3-II load and autophagic flux in PC12 cells [88]. Autophagic flux needs very careful fine-tuning to maintain neuronal homeostasis. On one hand, a basal level of autophagy is critical for neuronal homeostasis and survival [89] while on the other hand, the autophagy-inhibiting mTORC1 pathway is crucial for proper neuronal development [90,91]. 

Although autophagy is necessary for maintenance of cellular homeostasis and its inhibition has been shown to result in neurodegenerative phenotypes [92,93,94,95], its role in the acute cell stress response is not entirely characterized. Our findings show that even under constant inhibition of the autophagic process, we do not have an exacerbation of the oxidative stress response, indicating that autophagy is not a frontline protection against acute oxidative stress in differentiating neural precursors. In fact, evidence from animal models has shown that overactivation of autophagy can be detrimental to neurons and inhibition of autophagy has a neuroprotective effect under acute stress conditions [96,97,98,99,100]. However, to our knowledge human iPSC-derived systems have so far not been used to validate such findings. Moreover, a study in human HEK cells and *C. elegans* has shown that NRF2 inhibits autophagy under prolonged OS conditions and this has a protective effect on the cells [101]. This is in accordance with our finding that differentiated lt-NES cultures are able to resist TBHP-induced stress and maintain an active NRF2 pathway upon autophagy inhibition.

### 4.5. Proteasomal Degradation as a Key Regulator of the NRF2 Pathway Response under Oxidative Stress Conditions

Our proteasomal inhibition experiments indicate that the maintenance of the levels of NRF2 and KEAP1 protein is crucial for the proper functioning of the pathway rather than the absolute NRF2 protein abundance. In consequence, augmentation of proteasomal function could be a promising solution for inducing proper NRF2 function in neurodegenerative conditions. NRF2 has been identified to play a role in the oxidative stress response in Parkinson’s disease neurons [102]. In line with this, a recent study revealed that overexpressing NRF2 can be used to reverse alpha-synuclein pathogenic phenotypes [103]. Interestingly, the authors reported that NRF2 overexpression itself did not present an optimal solution since the effects of the overexpression were only transient. This is also in accordance with a study that showed that NRF2 activation is functioning in an oscillatory manner and does not depend on the levels of NRF2 protein but rather on its ability to shuttle in and out of the cell nucleus [104]. This notion is supported by data from another study where the ratio of NRF2 to KEAP1 protein levels in murine cells was found to remain low during physiological conditions with increased NRF2 nuclear translocation upon stress [105]. We see that accumulation of NRF2 itself, caused by temporary proteasomal inhibition, cannot keep the pathway active under oxidative stress conditions, which fits with these findings. It will be interesting to further analyze the mechanisms behind this and if NRF2 is blocked in the nucleus or the cytoplasm.

### 4.6. lt-NES Cultures as a Proxy for Human Neural Development In Vitro

In our experimental setup we detected the occurrence of a small fraction of astrocytes at later stages of maturation, which is a normal hallmark of neurodevelopment. Further studies will be necessary to explore the contribution of the glial population to NRF2 pathway activity and to delineate the precise interactions between neurons and glia in the context of controlling the response to oxidative stress. Importantly, at 6 weeks of differentiation (a time point characterized by a predominant neuronal population), we see metabolism-associated gene expression changes, NRF2 pathway activation and increased resistance to oxidative stress. Of course, this does not exclude an expected protective effect of glia on neurons in our model. In fact, glial cells are known to provide essential support for neurons, especially by exerting strong NRF2 pathway activity [106], and although they do not represent the predominant cell type in differentiated lt-NES cultures, they might still have an important protective effect on neurons. 

The supporting role of glia cells was already presented in neuronal differentiation of iPS cells, where culturing of iPS neurons in the presence of rodent primary astrocytes results in neurons with more mature phenotypes [82,107]. However, when TBHP toxicity was investigated in hippocampal rat neurons and astrocytes, TBHP induced the same effects on cell viability in both cell types [108]. This observation would suggest that the increasing resistance to stress observed during lt-NES differentiation is not primarily due to an emerging glial fraction. On the other hand, neurons and glia in rat hippocampal cultures have been shown to display different protein turnover rates with the protein turnover in neurons being affected by the presence of glial cells [109]. Therefore, one should not exclude the possibility that the neuronal NRF2 pathway displays different activation in the presence of astrocytes. Glial cells are an indispensable part of normal human nervous system development, promoting neuronal maturation, and, therefore, they are an important element in any in vitro model, aiming at recapitulating these events. Moreover, they also play a critical role in chemically induced mechanisms of neurotoxicity [110]. Importantly, rodent astrocytes were also observed to present more mature phenotypes upon neuron co-culture conditions [82]. These synergistic effects underpin the importance of a properly balanced mixed culture which contains both neurons and glia cells in order to model the in vivo situation in vitro. For future experiments, in order to decipher the individual contribution of each cell type in a physiologically relevant manner, analyses such as single cell RNAseq should be employed in mixed neuron-glia culture models, e.g., using lt-NES cultures differentiated for more than 6 weeks.

## 5. Summary and Conclusions

In conclusion, the human neural stem cell system employed here demonstrates the complex interactions of developmentally regulated pathways involved in modulating stress responses. In this context, the data from our reductionist model suggest that the interplay between the NRF2 pathway and proteasomal activity is crucial for the response to oxidative stress in the developing nervous system. The obtained data on the dynamics and activity of proteasomal function and autophagy across neuronal differentiation and their intersection with oxidative stress responses mediated via NRF2 signaling should provide a basis for delineating time windows suitable for studying pathway-specific developmental neurotoxicity and eventually preventative therapies for high-risk groups, exposed to DNT-inducing chemicals and other forms of prenatal stress.

## Data Availability

Further details on transcriptome raw data are available upon request.

## Supplementary Materials

The following supporting information can be downloaded at https://www.mdpi.com/article/10.3390/cells11091388/s1, Figure S1: Characterization of long-term lt-NES cell differentiation, Figure S2: Metabolic changes across lt-NES cell differentiation, Figure S3: NRF2-downstream target gene expression, Figure S4: ML385 treatment leads to decrease in NRF2 protein levels and affects cell viability of proliferative lt-NES, Figure S5: Extended blockade of autophagic flux in differentiated lt-NES cells, Figure S6: Characterization of 20S related proteasomal subunit β5 expression along lt-NES cell differentiation, Figure S7: 20S activity can be blocked by MG132 treatment, Figure S8: Autophagy induction upon MG132 treatment, Figure S9: Quantification of Western blot data of KEAP1 protein levels in lt-NES cells differentiated for up to 12 weeks treated for 17 h with MG132, Figure S10: The proteasome is regulating KEAP1-NRF2 pathway activation under acute oxidative stress conditions also in lt-NES cells differentiated for 6 weeks. Table S1: Inhibition concentration 20 (IC20) and 50 (IC50) value estimation from dose–response curve analysis of 24 h TBHP treatment presented in Figure 1D, Table S2: Primers, Table S3: Antibodies and stains.

## Abbreviations

ARE	antioxidant response element
DNT	developmental neurotoxicity
KEAP1	Kelch-like ECH-associated protein 1
NRF2	nuclear factor (erythroid-derived 2)-like-2 factor
OS	oxidative stress
OXPHOS	oxidative phosphorylation
ROS	reactive oxygen species
SNP	single-nucleotide polymorphism
TBHP	tert-butyl hydrogenperoxide
UPS	ubiquitin-proteasome system
hESC	human embryonic stem cells
iPS	induced pluripotent stem cell
lt-NES cells	long-term self-renewing neuroepithelial stem cells
hPSC	human pluripotent stem cell
