# Supplementary material for: Dynamics of Metabolic Pathways and Stress Response Patterns during Human Neural Stem Cell Proliferation and Differentiation
Source: Cells. 2022 Apr 20;11(9):1388. doi: 10.3390/cells11091388 (PMC9100042; doi:10.3390/cells11091388)
Supplement: Supplementary file 1 [file cells-11-01388-s001.zip › cells-1421445-supplementary.pdf]

Dynamics of Metabolic Pathways and Stress Response Patterns during Human Neural Stem Cell Proliferation and Differentiation

Vesselina Semkova [1,2], Simone Haupt [2], Michaela Segschneider [2], Catherine Bell [3], Magnus Ingelman-Sundberg [3], Mohamad Hajo [1], Beatrice Weykopf [1], Pathma Muthukottiappan [1], Andreas Till [1,*,†] and Oliver Brüstle [1,*,†]

[1] Institute of Reconstructive Neurobiology, University of Bonn School of Medicine & University Hospital Bonn, 53127 Bonn, Germany

[2] LIFE & BRAIN GmbH, Cellomics Unit, 53127 Bonn, Germany

[3] Karolinska Institute, Department of Physiology and Pharmacology, 171 77 Stockholm, Sweden

* Correspondence: a.till@uni-bonn.de (A.T.); brustle@uni-bonn.de (O.B.)

† These authors contributed equally to this work.

Abstract: Understanding early nervous system stress response mechanisms is crucial for studying developmental neurotoxicity and devising neuroprotective treatments. We used hiPSC-derived long-term self-renewing neuroepithelial stem (lt-NES) cells differentiated for up to 12 weeks as an in vitro model of human neural development. Following a transcriptome analysis to identify pathway alterations, we induced acute oxidative stress (OS) using tert-butyl hydroperoxide (TBHP) and assessed cell viability at different stages of neural differentiation. We studied NRF2 activation, autophagy, and proteasomal function to explore the contribution and interplay of these pathways in the acute stress response. With increasing differentiation, lt-NES cells showed changes in the expression of metabolic pathway-associated genes with engagement of the pentose phosphate pathway after 6 weeks, This was accompanied by a decreased susceptibility to TBHP-induced stress. Microarray analysis revealed upregulation of target genes of the antioxidant response KEAP1–NRF2–ARE pathway after 6 weeks of differentiation. Pharmacological inhibition of NRF2 confirmed its vital role in the increased resistance to stress. While autophagy was upregulated alongside differentiation, it was not further increased upon oxidative stress and had no effect on stress-induced cell loss and the activation of NRF2 downstream genes. In contrast, proteasome inhibition led to the aggravation of the stress response resulting in decreased cell viability, derangement of NRF2 and KEAP1 protein levels, and lacking NRF2-pathway activation. Our data provide detailed insight into the dynamic regulation and interaction of pathways involved in modulating stress responses across defined time points of neural differentiation.

Keywords: neurodevelopment; neuronal differentiation; NRF2; autophagy; ROS

Supplementary Information

Supplementary Figures

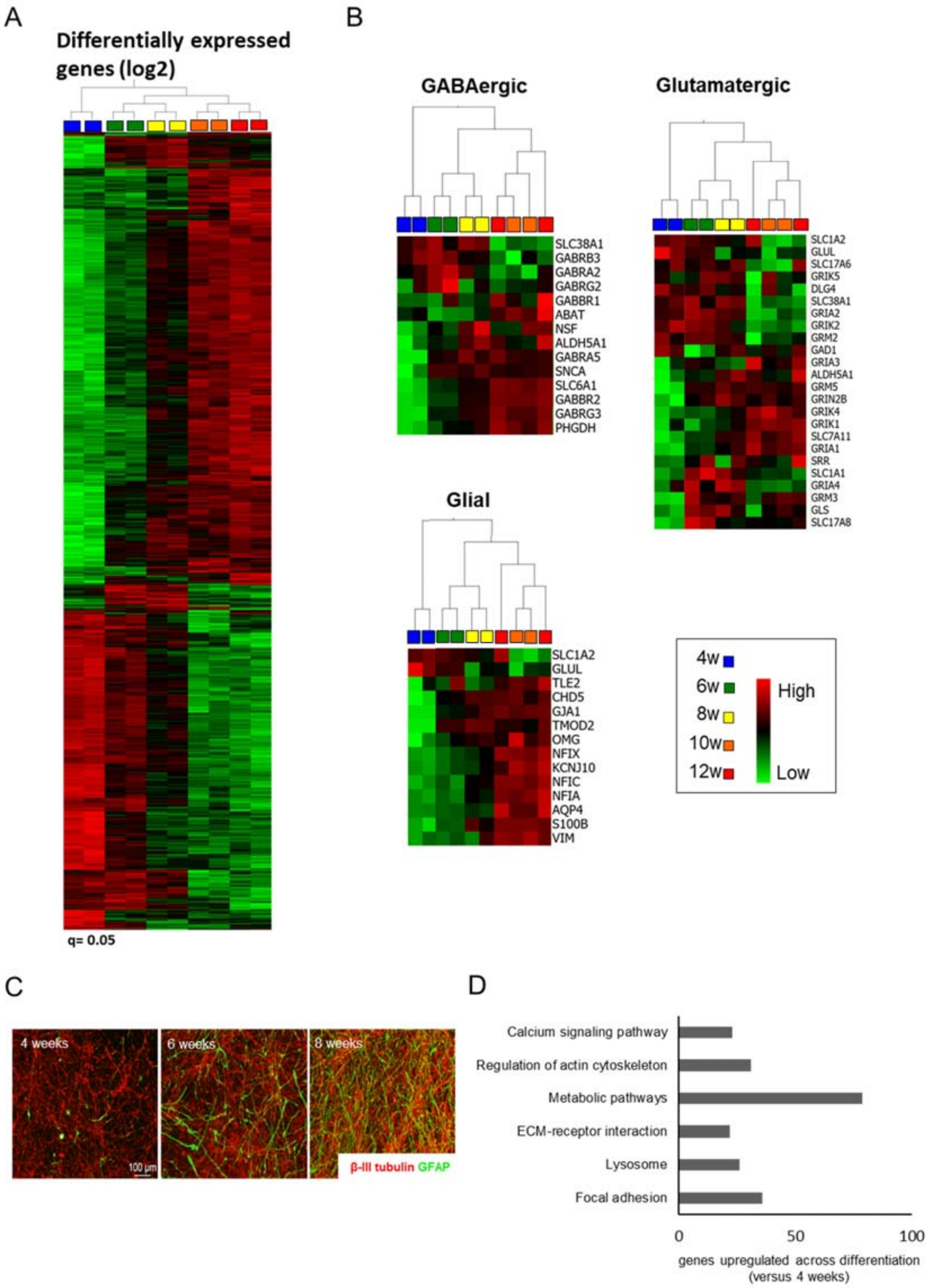

Figure S1. Characterization of long-term lt-NES cell differentiation. (**A**,**B**) Microarray-based transcriptome analysis of lt-NES cells across neural differentiation. (**A**) Heatmap of global

gene expression at 4, 6, 8, 10 and 12 weeks of in vitro differentiation. The two biological replicates analyzed per time point show prominent clustering according to differentiation time. Clustering analysis was performed by multigroup comparison, q = 0.05, n = 2. (**B**) Heatmaps depicting expression of selected gene sets associated with GABAergic and glutamatergic differentiation as well as glial fates across time of differentiation, n = 2. (**C**) Immunocytochemical analysis of lt-NES cells differentiated for 4, 6, and 8 weeks. Upon growth factor withdrawal, lt-NES cells give rise to a dominant fraction of neurons expressing β-III tubulin and a fraction of astrocytes positive for GFAP. Scale bar: 100 µm. (**D**) Gene Ontology Enrichment analysis of genes upregulated during lt-NES differentiation. Data were generated by comparing all gene expression profiles at time points > 4 weeks to the profile at 4 weeks (turning point of the differentiation trajectories) using an Affymetrix transcriptome microarray.

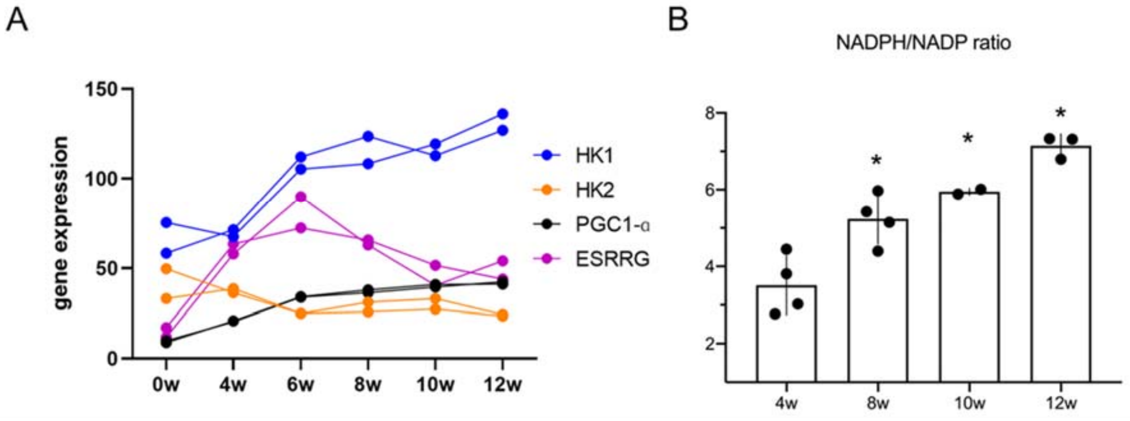

Figure S2. Metabolic changes across lt-NES cell differentiation. (**A**) Microarray-based analysis of basal expression of glycolysis (*HK1*, *HK2*) and mitochondrial biogenesis (*PGC1-alpha*, *ESRRG*) associated genes across lt-NES cell differentiation, pointing to a metabolic switch to OXPHOS characterized by switch from *HK2* to *HK1* at 6 weeks of differentiation. Significant differences across differentiation were found for several genes across differentiation, including *HK1*, *PGC1-α* and *ESRRG* for the comparison 0 weeks vs 6 weeks (n = 2 independent biological replicates). (**B**) NADPH/NADP ratios across differentiation. NADPH/NADP ratios were assessed in lt-NES cells differentiated for 4, 8, 10, and 12 weeks using NADPH-Glow assay (Promega). Statistical significance was assessed by one-way ANOVA (p = 0.0002) with Bonferroni's multiple comparison tests with the 4 weeks differentiation time point serving as control (4 w vs. 8 w: p = 0.0085; 4 w vs. 10 w: p = 0.0034; 4 w vs. 12 w: p < 0.0001); * = p < 0.05; n ≥ 2 per differentiation time point with 3 technical replicates for each biological replicate; each dot represents a biological replicate for the corresponding differentiation time point analyzed.

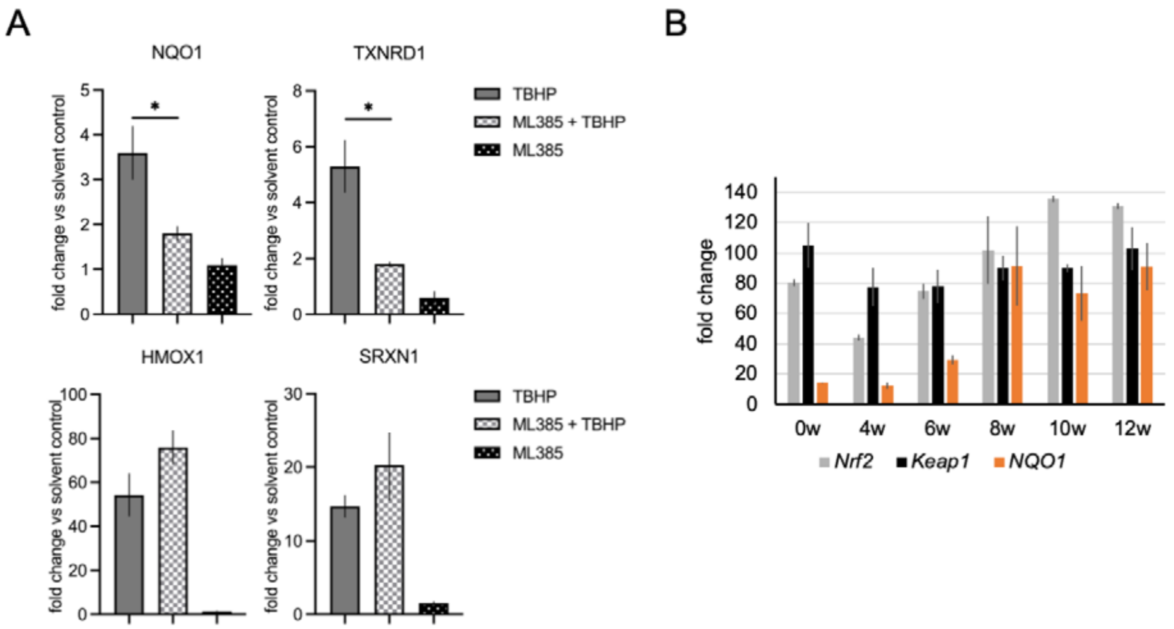

Figure S3. NRF2-downstream target gene expression. (**A**) qRT-PCR analysis of the specific NRF2 downstream targets *NQO1* and *TXNRD1* in lt-NES cells differentiated for 6 weeks upon treatment with the OS inducer TBHP alone or in combination with the NRF2 inhibitor ML385. Expression was normalized to GAPDH mRNA levels (after confirming stable expression of this reference gene across differentiation and treatments). Results are presented as means ± SEM in comparison to solvent control treated cells (equal to 1). Significant downregulation of target genes upon ML385 treatment was assessed by Student's t-test (* = $p < 0.05$); *NQO1* (n = 6; p = 0.0085), *TXNRD1* (n ≥ 3; p = 0.0016), *HMOX1* (n = 2), *SRXN1* (n = 2). (**B**) Analysis of basal *NRF2*, *KEAP1*, and *NQO1* gene expression levels in lt-NES cells across differentiation. Data were generated using an Affymetrix transcriptome microarray. Results are normalized to internal *GAPDH* mRNA levels; n=2 per differentiation time point.

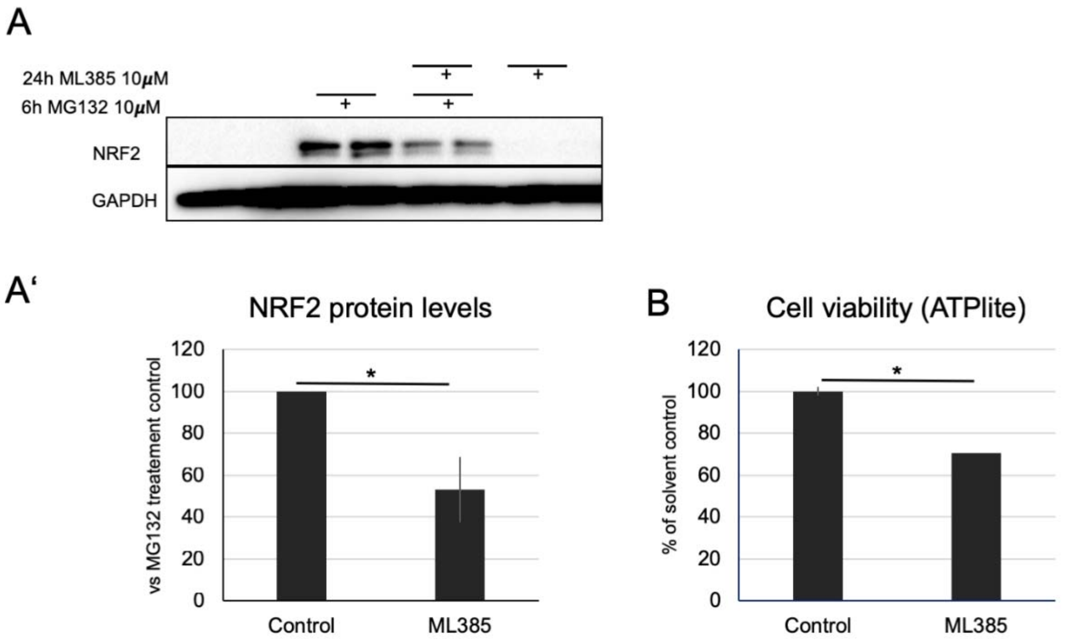

Figure S4. ML385 treatment leads to decrease in NRF2 protein levels and affects cell viability of proliferative lt-NES. (**A**) ML385 treatment is reducing NRF2 protein levels in lt-NES cells. WB of NRF2 protein levels in the presence of the NRF2 inhibitor ML385 with or without MG132. (**A′**) Quantification of immunoblot data from (**A**). NRF2 protein levels were evaluated as % of MG132 only treated samples. Statistical significance was assessed by Student's t-test ($p < 0.0001$); * = $p < 0.05$; n = 4. (**B**) ATPlite assay of lt-NES cells treated for 41 h with ML385. Statistical significance was assessed by Student's t-test ($p < 0.0001$); * = $p < 0.05$; n = 4.

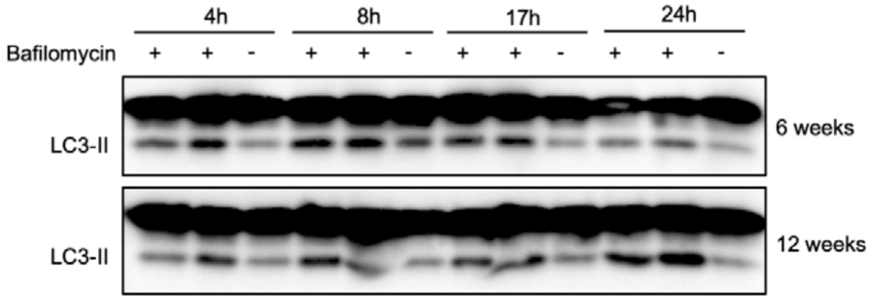

Figure S5. Extended blockade of autophagic flux in differentiated lt-NES cells. Autophagic flux assessment in lt-NES differentiated for 6 and 12 weeks and treated with Bafilomycin for 4 h, 8 h, 17 h, and 24 h and harvested for immunoblot analysis of LC3-II / LC3-I conversion.

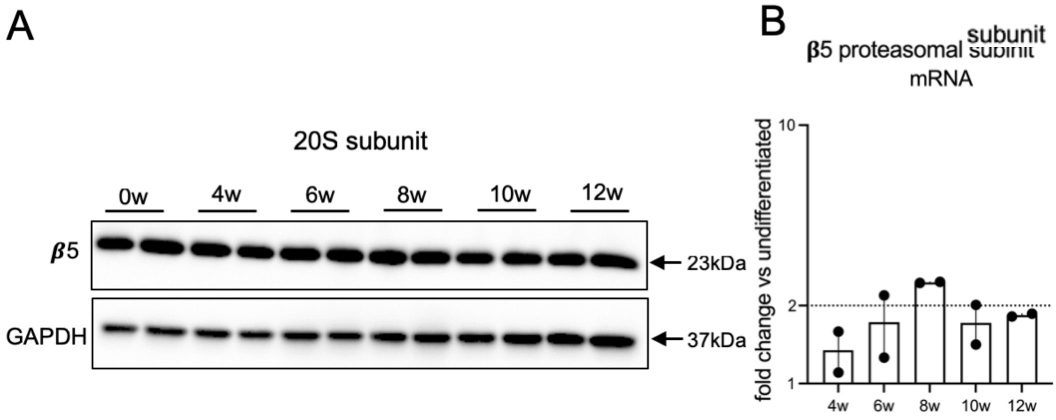

Figure S6. Characterization of 20S related proteasomal subunit β5 expression along lt-NES cell differentiation. (**A**) Representative immunoblot of 20S proteasome β5 subunit expression throughout lt-NES differentiation; n = 6 per differentiation time point. (**B**) qRT-PCR analysis of β5 subunit gene expression in lt-NES cells differentiated for up to 12 weeks. Results are presented in comparison to undifferentiated lt-NES cells (equal to 1). Data are normalized to *GAPDH* mRNA levels; n = 2 per differentiation time point; each biological replicate is indicated as a dot.

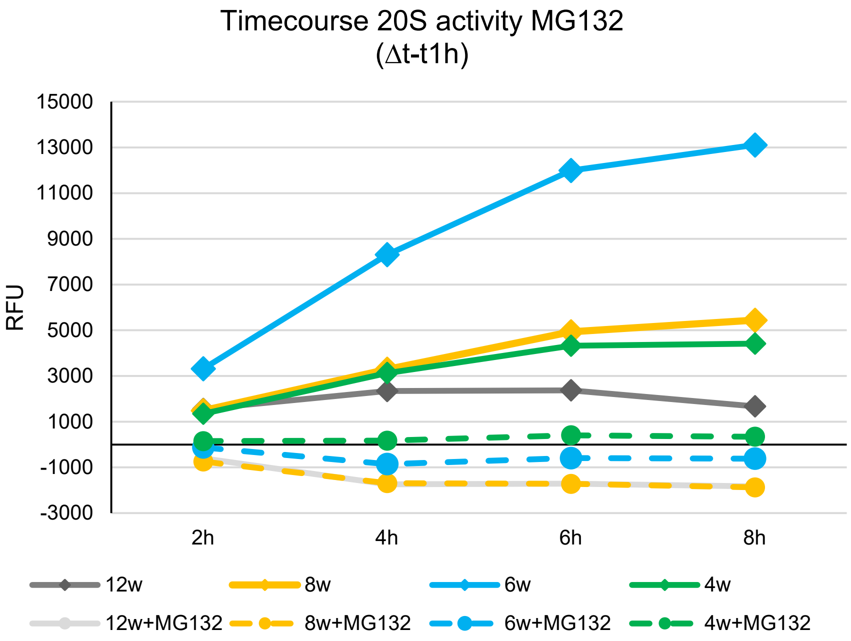

Figure S7. 20S activity can be blocked by MG132 treatment. 20S proteasome activity was measured in lt-NES cells differentiated for up to 12 weeks, using the proteasomal dye LLVY-R110. LLVY-R110 was added to the cells together with MG132 (10 µM) and fluorescence was measured after 1 h, 2 h, 4 h, 6h and 8 h. Proteasomal activity is presented as change in the fluorescence signal between each measurement time point and the baseline value at 1 h (Δt-t1h).

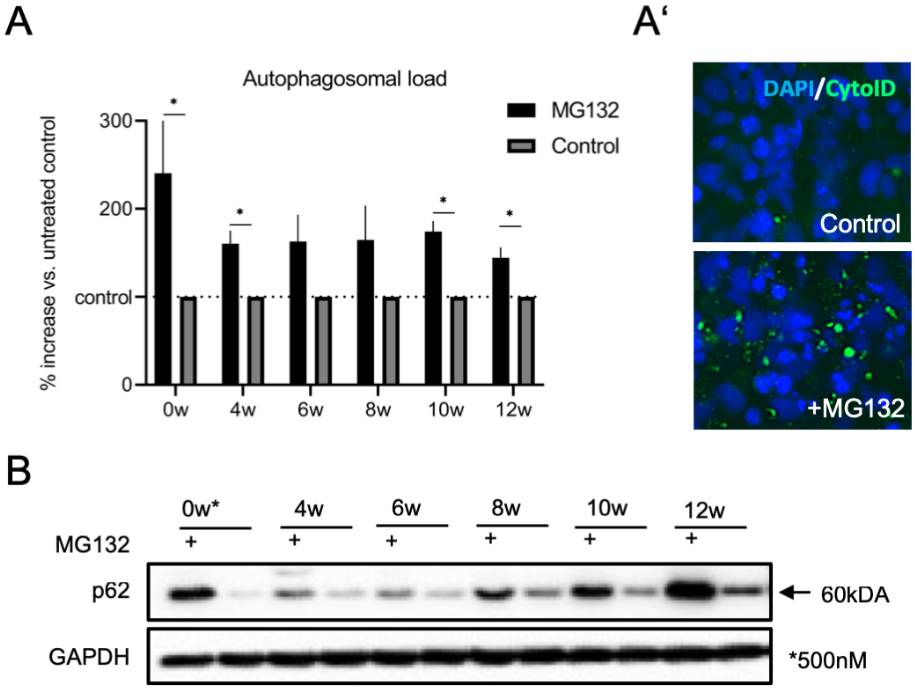

Figure S8. Autophagy induction upon MG132 treatment. (**A**,**A′**) (**A**) Autophagic flux response to MG132 (10 µM) treatment for 17 h in lt-NES differentiated for up to 12 weeks quantified by CYTO-ID® staining.

The autophagosomal load was assessed by calculating the ratio of autophagosomes to nuclei. Data are presented as % of untreated control for the corresponding differentiation time point ± SEM; n ≥ 3 per treatment and differentiation time point. Significant increase upon MG132 treatment was confirmed by unpaired Student's t test (* = $p < 0.05$). (**A'**) Representative CYTO-ID® staining images of lt-NES cells showing an increase in autophagosomal load upon MG132 treatment. Nuclei were counterstained with DAPI. (**B**) Immunoblot analysis of p62 across lt-NES differentiation after MG132 (10 µM; +) treatment for 17 h.

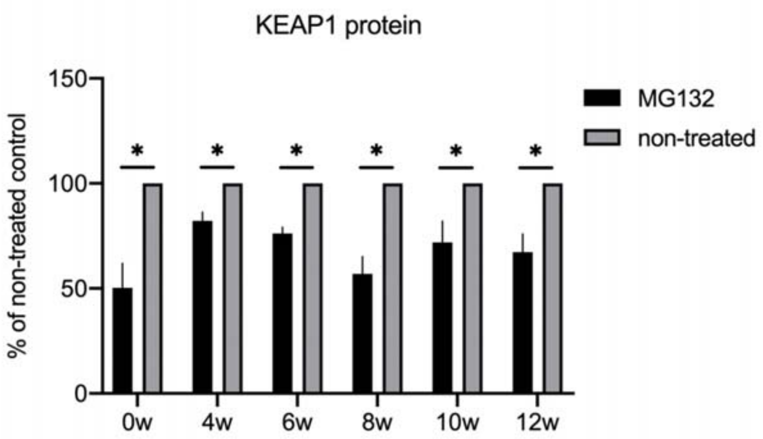

Figure S9. Quantification of Western blot data of KEAP1 protein levels in lt-NES cells differentiated for up to 12 weeks treated for 17 h with MG132. Data are presented as % of KEAP1 levels in untreated control cells of the respective differentiation time point ± SEM (n = 6). Significant difference between treatment and control was assessed by unpaired Student's t test (* = $p < 0.05$).

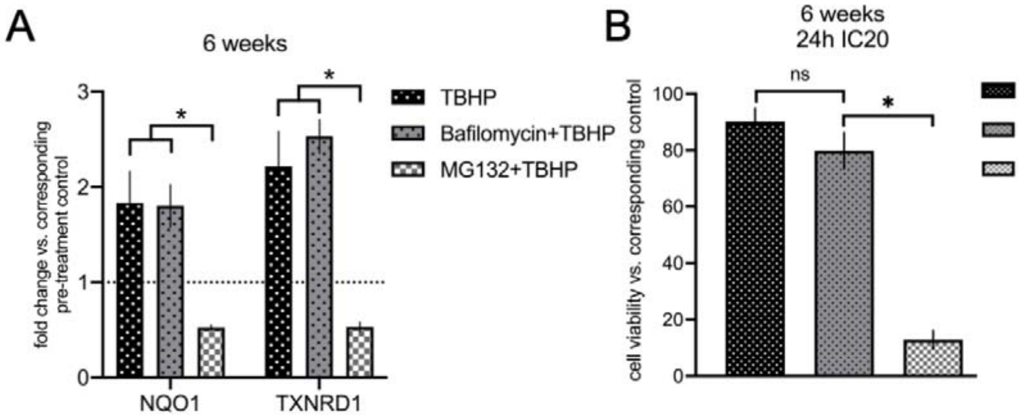

Figure S10. The proteasome is regulating KEAP1-NRF2 pathway activation under acute oxidative stress conditions in lt-NES cells differentiated for 6 weeks. (**A**) NRF2 pathway downstream target gene expression under OS induction upon inhibition of the proteasome (MG132) or the autophagic flux (Bafilomycin): Modulation of proteasome but not autophagy affects NRF2 downstream signaling. lt-NES cells were differentiated for 6 weeks and treated with TBHP for 8 h. Expression of the NRF2 downstream target genes *NQO1* and *TXNRD1* was analyzed by QRT-PCR. Results are presented in comparison to the corresponding pre-treatment condition (MG132, Bafilomycin or no pre-treatment control; equal to 1) ± SEM. Data are normalized to *GAPDH* mRNA levels. Statistical significance between means was assessed by Student's t-test (* = $p < 0.05$); n = 4; MG132+TBHP condition vs. TBHP condition: *NQO1* p=0.0072; *TXNRD1* p=0.0036. (**B**) Effect of proteasomal or autophagic inhibition on the ability of lt-NES cultures differentiated for 6 weeks to cope with TBHP toxicity. Cells were pre-treated with MG132 or Bafilomycin followed by a 24 h TBHP treatment. Cell viability was assessed at the end of the treatment by ATPlite

assay. Results are presented as percentages relative to untreated cells (equal to 100 %). Data are presented as means ± SEM (n = 4, consisting of at least 4 technical replicates each). Significant difference between means was assessed by Student's t-test (* = $p < 0.05$); n = 4; Bafilomycin+TBHP condition vs. TBHP condition: $p = 0.2242$; MG132+TBHP condition vs. TBHP condition: $p < 0.0001$.

Supplementary Tables

Table S1. Inhibition concentration 20 (IC20) and 50 (IC50) value estimation from dose-response curve analysis of 24 h TBHP treatment *presented in Figure 1D. CI, confidence interval.*

**Differentiation time**	**IC50 (µM)**	**IC20 (µM)**	**(95 % CI) µM**
0 weeks	101	62	(55–70)
4 weeks	134	72	(55–90)
6 weeks	293	180	(142–222)
8 weeks	402	290	(240–343)
10 weeks	1184	727	(551–971)
12 weeks	1099	800	(587–906)

Table S2. Primers

**Target gene**	**Forward**	**Reverse**
GAPDH	GGCCTCCAAGGAGTAAGACC	AGGGGTCTACATGGCAACTG
HMOX1	AAGACTGCGTTCCTGCTCAA	TCTTGCACTTTGTTGCTGGC
NQO1	AGGACCCTTCCGGAGTAAGAA	TGGAAGCCACAGAAATGCAGA
PSMB5 (β5)	GGGAGTCTCAGTGATGGTCTG	GGCGGAACTTGAAGGCCA
SQSTM1 (p62)	AGAATCAGCTTCTGGTCCATCG	TTCTTTTCCCTCCGTGCTCC
SRXN1	GATCCGGGAGGACCCAGACA	CAAGGAGGCTGCTACTGCAA
TXNRD1	ATGTCATGTGAGGACGGTCG	TCTGCCCTCCTGATAAGCCT

Table S3. *Antibodies / Stains*

**Antigen / Conjugate**	**Origin**	**Dilution**	**Provider**
GAPDH	mouse IgG	1:1000	Santa Cruz Biotechnology, Heidelberg, Germany
GFAP	rabbit IgG	1:1000	Thermo Fisher Scientific, Braunschweig, Germany
KEAP1	mouse IgG	1:500	Santa Cruz Biotechnology, Heidelberg, Germany
LC3B	mouse IgG	1:2000	Enzo Life Science, Lörrach, Germany
MAP2	mouse IgG	1:200	SIGMA-ALDRICH/MERCK, Darmstadt, Germany

NFE2L2	rabbit IgG	1:200	Cell Signaling Technology Europe, Frankfurt am Main, Germany
NQO1	mouse IgG	1:500	Santa Cruz Biotechnology, Heidelberg, Germany
PSMB5 (β5)	mouse IgG	1:500	Santa Cruz Biotechnology, Heidelberg, Germany
SQSTM1/p62	rabbit IgG	1:1000	MBL Life Science, Nagoya, Japan
TXNRD1	mouse IgG	1:500	Santa Cruz Biotechnology, Heidelberg, Germany
β-III Tubulin	mouse IgG	1:1000	Covance, Münster, Germany
Alexa Flour 488 / 555	rabbit IgG	1:1000	Thermo Fisher Scientific, Braunschweig, Germany
Alexa Flour 488 / 555	mouse IgG	1:1000	Thermo Fisher Scientific, Braunschweig, Germany
HRP	mouse / rabbit IgG	1:1000	Cell Signaling Technology Europe, Frankfurt am Main, Germany
Cell Nuclei	DAPI	1:1000	Thermo Fisher Scientific, Braunschweig, Germany
Cell Nuclei	Hoechst	1:1000	Enzo Life Science, Lörrach, Germany
